# Effective leaders(hip) in community-academic health partnership projects: An inductive, qualitative study

**DOI:** 10.3389/fpubh.2022.941242

**Published:** 2022-08-12

**Authors:** Choiwai Maggie Chak, Lara Carminati

**Affiliations:** ^1^Science-to-Business Marketing Research Centre, FH Münster University of Applied Sciences, Münster, Germany; ^2^Faculty of Behavioural, Management and Social Sciences, University of Twente, Enschede, Netherlands

**Keywords:** community-academic health partnership, effective project leadership, Grounded Theory, qualitative research, thematic analysis

## Abstract

To deepen our understanding of how project leaders can lead effectively in different community-academic health partnerships (CAHPs), we conducted an inductive, qualitative study through semi-structured interviews (*N* = 32) and analyzed the data with Grounded Theory approaches. By presenting a process model illustrating the cycle of effective leaders(hip) in CAHP projects, we contribute to the literature on CAHP, leadership development, and complexity leadership theory in three ways. Firstly, the model depicts the strategies enabling leaders to navigate typical project challenges and perform leadership tasks effectively. Secondly, we distill four beneficial qualities (i.e., adopting a proactive attitude, having an open and adaptive mindset, relying on peer learning and support, and emphasizing self-growth and reflexivity) which CAHP project leaders require to develop themselves into effective leaders. Thirdly, we illustrate leaders' dynamic developmental logics and processes of effective leadership and their contributions to better project functioning in diverse CAHPs.

## Introduction

Nowadays, public health challenges such as drug addiction, obesity and physical inactivity are increasingly addressed through community-academic health partnerships (CAHPs) (1). In a CAHP, academic researchers actively include and recombine diverse community stakeholders' knowledge, resources, and capacities to generate rigorous research and/or targeted health interventions and innovations (2). However, CAHPs addressing such wicked health challenges are often intrinsically complex, networked systems that are resource-intensive to manage (3, 4). Moreover, their successes depend heavily on the dynamic interplay between community and academic partners (5, 6). Growing literature has pointed to the decisive role of effective leadership in orchestrating such complex dynamics (7–9) and steering the partnerships toward successful and sustainable outcomes (4, 10).

Nevertheless, such outcomes are often undermined by numerous challenges that CAHP project leaders constantly need to tackle when bringing diverse stakeholders together for the common purpose of the project (11). These challenges can hinder their ability to perform project leadership tasks effectively (12). For example, beyond the daunting duty of securing project resources and reaching goals (13), they often need to operate in ambiguous leadership roles (14), act in uncertain environments (15) and must manage the unavoidable conflicting interests or demands between the diverse partnership members (8). Nevertheless, only a few concrete field studies have illustrated *how* project leaders address such complex challenges in different CAHP settings (13, 16). As a result, how project leaders pursue effective leadership sustainably in diverse CAHPs remains largely unexplored (5, 15).

This knowledge gap can be attributed to two main reasons. Firstly, most studies have been criticized for reporting only on the effectiveness of specific health interventions and accomplishments whilst neglecting to include details of any struggles, unsuccessful attempts, and useful strategies or processes employed in response to these challenges (13, 17). Secondly, despite the recognized significance of leadership on CAHP effectiveness in the literature, there were considerable variations and ambiguities in how scholars conceptualize “leadership” (18). For example, some studies have considered leadership as individual leaders' traits or characteristics (14); others have examined more distributed forms of leadership, such as collaborative leadership (19), collective leadership (20) and shared leadership (21). The inconsistencies in leadership conceptualization, coupled with the overlooked dynamics and impacts of CAHP project settings on leadership practices, have precluded scholars from drawing answers on how effective leadership and leaders, from decision-making to strategic issues, jointly contribute to effective CAHPs (7).

Hence, to examine how leaders can perform their leadership functions and roles sustainably and effectively in complex CAHP systems (8, 22), a more focused perspective accounting for both effective *leadership* and effective *leaders* is required (23). Additionally, CAHP scholars have called for empirical work to obtain a more nuanced and thorough understanding of the complex inner workings of project implementation (24) and leaders' efforts in handling the dynamics in different CAHPs (11). To this end, a growing body of health care research has proposed to examine the interplay of project leaders' behaviors under varied contextual forces (e.g., actors, challenges, and contexts) through the lens of Complexity Leadership Theory (CLT) (22).

Complexity leadership theorists posit that a triadic model of operational, enabling and entrepreneurial leadership behaviors allows leaders to unite diverse perspectives and create shared values in collaboration (25). This theory further complements extant leadership research by highlighting the critical role of environmental dynamics on leaders' actions (26) and bringing greater attention to the facilitative mechanisms and processes for better learning, innovation, and adaptability in CAHPs (27). However, CLT falls short in three aspects in explaining how CAHP projects can be led effectively. Firstly, although CLT provides a meta-framework for leadership behaviors at the organizational level (25), it remains conceptually abstract and lacks empirical descriptions of the strategies for addressing the specific challenges in diverse interorganizational, networked settings like CAHPs (23, 28). Secondly, the theory has not offered much guidance on becoming a better leader in complex, networked project environments (25). Thirdly, how leadership and leaders evolve and contribute to desirable outcomes in complex systems like CAHPs remains largely unexplored (22).

Independently, both CAHP and CLT scholars have called for qualitative research to offer richer insights into project leaders' notions of effective leadership (20, 29), particularly on strategies and qualities that enhance leaders' readiness and ability to excel in complex, networked systems (30, 31). Thus, to deepen our limited understanding of effective leadership and leading in different CAHP contexts and in an effort to fill some gaps in CLT, we embarked on a study to address the research question:

*How do project leaders perform their leadership functions and roles effectively in complex CAHP systems?*

We adopt an interpretivist approach to explore project leaders' subjective lived experiences and perceptions of effective CAHP leadership and leading. This study aims to contribute to the burgeoning CAHP and leadership research in three ways. Firstly, by exploring the inner workings of CAHP projects, we aim to unpack CAHP project leaders' practical strategies for navigating the challenges while performing *leadership* tasks effectively in CAHPs and similar complex network settings. Secondly, we aim to advance leadership development by exemplifying the beneficial qualities that project leaders should possess to become effective *leaders* in CAHPs. Thirdly, we aim to extend CLT by depicting the dynamic developmental logics and processes of effective *leadership* and *leaders* in a CAHP project and their contributions to enhanced project functioning.

## Materials and methods

### Study design

We conducted an inductive, qualitative inquiry with leaders from diverse CAHP projects in Germany to explore their lived experiences in leadership and leading. By conducting semi-structured key informant interviews, we aimed to capture the characteristics of effective leadership and leaders based on their past efforts to address the challenges that arose in their projects. This qualitative method provides a rich and detailed description of the often-neglected inner workings of CAHP project leadership with a focus on differentiating between the characteristics of effective *leaders* and those of effective *leadership*.

### Recruitment and sample characteristics

In the absence of a complete, updated list of all German CAHP projects, we were unable to generate a comprehensive sampling frame for random sampling (pp. 298) (32). Therefore, we adopted a heterogeneous purposive sampling strategy (pp. 337) (32) and compiled a sampling frame based on active web searches to identify eligible CAHP projects (e.g., project websites and participatory project networks). The key terms used for searching were: (“patient^*^ *OR* “community^*^” OR “societ^*^”) AND (“universit^*^” OR “academic” OR “research^*^”) AND (“alliance^*^” OR “collaborat^*^ OR “participatory” OR “partners^*^”) AND “health”). As inclusion criteria, eligible CAHP projects were identified based on (1) definition of a community-academic partnership: a collaborative relationship between at least one researcher and at least one community member(s) (i.e., representative or agency) from the field(s) of business, health care organization, policymaking, or civil society (e.g., nongovernmental organizations, churches, charities, schools); and specific health-promotional cause(s) that is/are relevant to the community of interest. To reduce the chances of recall bias, we only considered ongoing or recently completed CAHP projects between 2019 and 2021. Any projects that did not clearly describe their projects' causes, partners involved, or the relationships between community and academic partners were excluded. To ensure a broad range of perspectives, project leaders of eligible CAHP projects were selected regardless of their gender, experiences in CAHP project leadership, and backgrounds. Eligible project leaders were invited to participate in an interview *via* email. A reminder email was sent to the nonrespondents 1 week later.

Of the 137 formal CAHP project leaders invited, 32 participated in the study (23%). Thirteen (9.5%) of the invited leaders rejected the invitation due to unavailability (*N* = 10, 7.3%), retirement (*N* = 1, 0.7%), or being occupied with pandemic-related work (*N* = 2, 1.5%). Four contacts were no longer accessible (2.9%), while no replies were received from others after the reminders were sent (*N* = 88, 64.2%). Meanwhile, twenty-one of the participants were women, and eleven were men. All of them worked on entirely different projects. A detailed overview of each study participant and their CAHP projects is provided in Supplementary material 1. Interviewees were 49 years old on average (29 – 68 years old), with an average of 11 years of experience in CAHP project leadership (SD = 5.66). A majority of them also had a job position affiliated with a research institute or university (62.5%, *N* = 20), followed by (university) hospitals (12.5%, *N* = 4), government authorities (9.38%, *N* = 3), nongovernmental organizations (9.38%, *N* = 3), business/industries (6.25%, *N* = 2) and insurance companies (3.13%, *N* = 1). The thematic focuses of the CAHP projects in which interviewees were involved were diverse, ranging from health treatment/care improvement (*N* = 12), community health promotion (*N* = 10), education/training for health professionals (*N* = 4), patient support (*N* = 3), disease management (*N* = 2) to disease prevention (*N* = 1). The average duration of the projects was 4.5 years (SD = 3.54) (Table 1).

**Table 1:** Participant characteristics (*N* = 32).

Gender (%)	Women	21 (65.6 %)
	Men	11 (34.3 %)
Age [Mean (Range)]		49 (29-68)
Years of experience in project leadership [Mean (SD)]		11 (5.66)
Project duration in years [Mean (SD)]		4.5 (3.54)
Project leaders' affiliation (%)	Research institute/university	20 (62.50%)
	(University) hospital	4 (12.50%)
	Government authority	3 (9.38%)
	Nongovernmental organization	3 (9.38%)
	Business/Industry	2 (6.25%)
	Insurance company	1 (3.13%)
Education level (%)	Professorship	11 (34.38%)
	Doctorate	11 (34.38%)
	Postgraduate	6 (18.75%)
	Undergraduate	3 (9.38%)
	Diploma	1 (3.13%)
Project theme (%)	Treatment/care improvement	12 (37.50%)
	Community health promotion	10 (31.25%)
	Education and training for health professionals	4 (12.50%)
	Patient support	3 (9.38%)
	Disease management	2 (6.25%)
	Disease prevention	1 (3.13%)
Project funding source (%)	Federal funding	13 (40.63%)
	State/Regional funding	11 (34.38%)
	Insurance company	5 (15.63%)
	Private funding	3 (9.38%)
	European funding	2 (6.25%)
	Membership fee	1 (3.13%)
	Bank	1 (3.13%)

### Research instrument

A semi-structured interview protocol was developed and piloted with three project leaders from different CAHPs in Germany, ranging from health care management and health care education to disease prevention. The content of the interview protocol was then revised based on the interviewees' feedback to ensure the appropriateness, clarity, and comprehensibility of the questions (33). The final interview protocol comprised open-ended questions covering five main themes: project structure, leadership and decision-making processes, reflections on any (leadership) challenges, enablers, and performance in the projects. Interviewees were asked to describe the objectives and structure of their current or recently completed CAHP projects (e.g., “Could you please briefly describe the project?”); their previous experiences in leading any CAHP projects (e.g., “Have you also led/managed similar project(s)?”); their project roles and tasks (e.g., “How would you describe your role in the project?”); and the decision-making processes in the projects (e.g., “How are major decisions made in the project?”). Then, they were invited to illustrate if they had faced any significant challenges in leading the projects and to reflect on how they dealt with those challenges (e.g., “Have you faced any major setbacks/challenges in this project? How did you react to them?”). We also asked interviewees to note any enablers, strategies, or tactics that helped them address those challenges and evaluate their current projects' overall performance (e.g., “What have you found to be important in helping you (or your team members) cope with the challenges?”) (Supplementary material 2).

We implemented semi-structured interviews since they were deemed appropriate for deeper probing into participants' perception of effective leadership and leading practices and facilitating the identification and constant comparison of themes (34). All interviews were conducted digitally (*N* = 27) or *via* phone (*N* = 5) between March 2020 and April 2021, following the safety regulations during the COVID-19 pandemic. Interviews were conducted in German or English, audio-recorded and transcribed verbatim by native speakers. German transcripts were then translated into English by fluent bilinguals. The interviews lasted between 30 and 60 min, yielding 382 single-spaced pages for data analysis.

The study was approved by the Ethics Committee of the University and complied with the General Data Protection Regulation. We obtained verbal and written consent from all interviewees before the interviews and reassured them that their participation was voluntary, strictly confidential, and anonymous. Considering the interviews were conducted digitally or *via* phone and that the accuracy of transcripts could potentially be affected by any background noises or technical issues, all transcripts were sent back to interviewees for corrections or additional comments. Transcripts were anonymized to conceal participants' identities and personal information after receiving interviewees' potential corrections or comments.

### Data collection and analysis

We followed (34, 35) suggestions and analyzed the data in parallel with the data collection process. After each of the three interview rounds (March–April 2020; October–November 2020; and March–April 2021), we performed preliminary analyses to obtain initial insights and identify knowledge gaps. The interview protocol was then revised as the research progressed to identify the themes concerning our research questions (35). We then collected and analyzed the data iteratively until we reached theoretical saturation, when no new insights emerged from adding further study participants (36).

**Figure 1:**
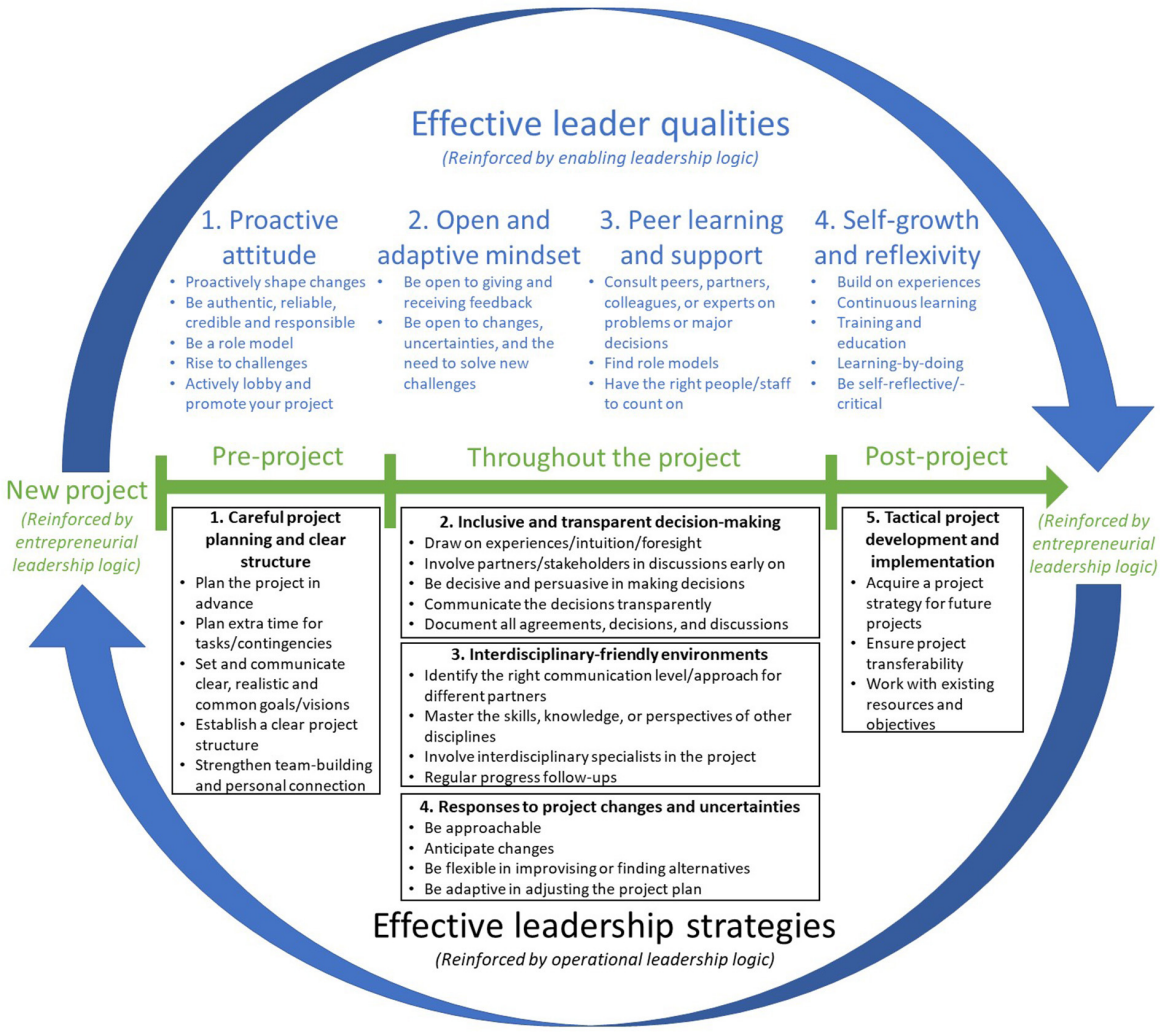
Effective CAHP project leaders(hip) cycle.

Using Corbin and Strauss' Grounded Theory approaches (34) and Gioia et al. inductive coding process (35), two bilingual coders analyzed the transcripts and performed the initial inductive coding process separately. Here, open codes adhering to the terms and expressions used by interviewees were generated (34). During the process, the coders also performed memo writing, in which notes and observations were written, sorted, and resorted to offer a firm base for theoretical development (34). Findings were then constantly compared, discussed, and refined between the coders until a consensus on data interpretation was reached (35). Subsequently, the coders discussed any themes or insights derived from the data and performed axial coding, a process in which relationships among open codes (i.e., first-order concepts) were identified to form sub-categories (i.e., second-order themes) after constantly testing the linkages proposed against the data collected (34, 35). This process gave rise to the theory-centric, second-order themes, which enabled us to explore the relationships among the first-order concepts and eventually to cluster the themes into three aggregated dimensions relevant to our research questions (35). The analysis was carried out using the MAXQDA 2020 software. We recursively referred to the collected data, emerging insights, and extant literature to establish linkages between the identified themes. We then synthesized the findings and constructed a process model depicting the cycle of effective CAHP leaders(hip) (Figure 1).

## Results

While our primary focus was to answer how project leaders could perform their leadership functions and roles effectively in complex CAHP systems, we present the leadership challenges faced by project leaders as part of our findings to provide a better contextual reference for elucidating the complex realities of leading CAHPs. Accordingly, three overarching themes emerged: (a) leadership challenges faced by CAHP project leaders; (b) effective leadership strategies for dealing with those challenges; and (c) effective leader qualities. The data structures for all themes are shown in Supplementary material 3. Illustrative quotes are presented with pseudonyms to protect interviewees' identities, along with their age and years of experience in CAHP project leadership (Y.o.E). Additional responses coded to each theme are summarized in Supplementary material 4.

### Leadership challenges faced by CAHP project leaders

Five second-order themes emerged concerning the leadership challenges interviewees encountered while leading their CAHP projects: project planning and management, the balance of participatory decision-making, project interdisciplinarity, project changes and uncertainties, and lacking project impacts and sustainability.

### Challenge 1: Project planning and management

In CAHP projects, planning adequate time and resources for project task execution was a common challenge for relatively inexperienced CAHP project leaders (<5 years of experience). For example, one of the interviewees underlined that sometimes they happened to be under-resourced due to unforeseen expenses on some project tasks: “*In some cases, we applied for too little [funding]. For example, in a training course, we did not consider some of the interviews still have to be translated, that we somehow need funds for translators.” (Jasmine, Age 35, 2 Y.o.E)*

In the same vein, many interviewees mentioned the complexity of defining and clarifying project management roles and responsibilities in a highly decentralized project setting. For instance, they must first take the time to understand the specific structural conditions and differences among the partner members and their institutions to define their roles and responsibilities in the projects:

“*At the beginning, it took a lot of discussion for all of us to realize that there is external project management, which is my responsibility; and internal project management, which partners lead a bit like the scouts from different institutions - as they cannot always turn to their original institution when there are things to be clarified for the project*. *It's like a separate institution where you work together without having the same employer*.”* (Sophie, Age 54, 9 Y.o.E)*

Sometimes, they also had to be familiar with new requirements or structures and help partners understand and deal with them. For example, a respondent noted it was challenging for him to get familiar with legal topics and to manage the finance:

“*The most difficult thing for me was…to implement the project and to draft it in a way that it would be legally sustainable…I have no idea about the law…” (Moses, Age 56, 2 Y.o.E)*

Accordingly, they often had to tailor their leadership approaches due to the different requirements, organizational structures, project team compositions and working styles of partners and their organizations in each project. One of the participants noted: “*For every project, everything you lead is different. And you'll have to get to know the people that are working on it and in it.” (Janet, Age 31, 3 Y.o.E)*.

Due to the uniqueness of each project setting, leaders must devote extra time to discuss with the project partners, understand how specific structural and environmental dynamics may impact their project planning and implementation and explore the most effective ways of leading.

### Challenge 2: The balance of participatory decision-making

Several interviewees mentioned that their projects adopted a high degree of participatory or shared decision-making processes, where decisions were mostly or always made by consensus among project partners. For example, a respondent mentioned: “*So, there is no hierarchy in the sense that someone has the authority to give orders, but everything* ***only*** *[emphasised] works by consensus.” (Moses, Age 56, 2 Y.o.E)*

However, a few interviewees also struggled to determine the “*right mixture of participation and leadership” (Iris, Age 35, 2 Y.o.E)* in their projects and to channel the information to suit partners' desired level of engagement. For instance, a project leader explained that although decisions about project content were always made collectively, she recognized that it is sometimes impractical to adopt a fully participatory or shared leadership style in a large-scale project with remote partners, since the communication process could become time-consuming and strenuous, eventually leading to partner disengagement:

“*At the beginning, I really asked a lot of questions in the round and tried to decide together, which was very difficult with the number of consortium partners and also the distance... This unfortunately made you realize that certain things simply had to be decided by yourself… you can't give all decisions to everyone because it doesn't lead to consensus. Now many people no longer participate in the decisions. There is no feedback.” (Claire, Age 40, 6 Y.o.E)*

It is clear that many project leaders struggled to find the balance between participatory and unilateral decision-making, as they had to adapt quickly to partners' feedback, determine when to make decisions collectively, and adjust their leadership strategy when necessary to keep the project moving.

### Challenge 3: Project interdisciplinarity

Despite years of experience leading CAHP projects, harmonizing the diverse perspectives and satisfying the varied needs and interests among partners remained challenging for some project leaders due to the interdisciplinarity in their projects. One of them highlighted:

“*So, I think that is a challenge… especially when it comes to public health in this project, then you are suddenly in a broad field where quite a lot of perspectives come together: the medical perspectives, the psychological, sociological, and communicative perspectives…and I also find it not quite easy to orient oneself there.” (Barry, Age 64, 9 Y.o.E)*

Ensuring effective interdisciplinary communication was also a tremendous hurdle for a few interviewees. According to one of them, for instance, interdisciplinary scientists often “*cannot get into the heads of the others*” *(Bonnie, Age 35, 2 Y.o.E)*. Communication became more complicated while leading in the absence of hierarchy, for which leaders must be open to opinions from all sides. Meanwhile, they must also exert their influence on project members to attain the intended goals: “*I don't have any disciplinary responsibility above anyone. This means that I cannot claim a managerial position… I must try to exert influence on other project members, for example, to be able to achieve the goals.” (Ron, Age 26, 2 Y.o.E)*

As a result, project leaders had to orient themselves to accommodate partners' diverse perspectives and deal with issues concerning interdisciplinary and interorganizational communication.

### Challenge 4: Project changes and uncertainties

Since many project activities were affected by external influences such as the COVID-19 pandemic, some project leaders reported facing a high degree of uncertainty in their projects. One of them underlined:

“*There was a great deal of uncertainty about how things would continue as a team here…about what to do now…We have, of course, adjusted some of the goals, maybe even reduced them…because the expectation was that we would catch up after the lockdown. But it is not that easy.” (Lily, Age 34, 2 Y.o.E)*

Sometimes due to uncontrollable external influences (e.g., change in political will), project leaders were forced to adjust their project direction or even discard the projects. A respondent noted:

“*If there are external influences, where you conclude that the vision has to be changed, or maybe it has to be discarded, or the project ends for this; that is, of course, a manslaughter. Nothing can be done about that…you have to look for alternatives or go in a completely different direction and redefine it completely.” (Elaine, 42, 4 Y.o.E)*.

Hence, the need to promptly react to the external changes and uncertainties to adjust or cut back on project goals, as well as to change plans while in progress, could lead to worries and stress about achieving the intended project goals on time.

### Challenge 5: Lacking project impacts and sustainability

Some project leaders commented on the lack of impact and sustainability in their projects due to uncontrollable external influences, such as limited funding or project duration and regulation changes, forcing them to seek new projects. For example, an interviewee expressed: “*I would say that the project needs to grow more. And the problem is that it will only be there for four years and then it is gone. There's no continuity.” (Carla, Age 49, 5 Y.o.E)*

It could also be demanding for projects that address controversial or unfamiliar topics to the public to gain enough societal support or acceptance to sustain themselves: “*The biggest challenge is to convince the funds because people don't understand what [the project topic] is.” (Anna, 53, 12 Y.o.E)*

Consequently, ensuring projects' acceptance, societal impacts, and sustainable outcomes could be challenging for some project leaders. Indeed, a lack of these elements could trigger additional difficulties in project execution (e.g., financial challenges) and threaten partnership sustainability.

### Effective leadership strategies

Five second-order themes were identified regarding the effective strategies adopted or suggested by interviewees to deal with the aforementioned challenges. They included: careful project planning and clear project structure; inclusive and transparent decision-making; creating interdisciplinary-friendly environments; responses to project changes and uncertainties; and tactical project development and implementation. These strategies are presented chronologically according to participants' suggested time of relevance in a project cycle (Figure 1).

### Strategy 1: Careful project planning and clear structure (pre-project)

In response to the challenges of having inadequate time and resources for project task execution, a few interviewees with prior experiences in similar projects highlighted the significance of careful project planning and better preparation *in advance* (i.e., as early as the project application stage), such as planning a buffer for time-consuming project tasks. For example, an interviewee mentioned: “*I know how often such an analysis goes wrong, and I can build that into the project planning. That works.” (Helen, Age 50, 10 Y.o.E)*

Apart from formulating and discussing the shared vision with partner members continuously, some interviewees also found it critical to establish a clear project structure *at the start of the project*. A predefined project structure can play a strategic role in facilitating the decision-making process and settling the differences, such as varied ways of working: “*You really get a structure in place and come to a decision, with all the differences that you might have in the team.” (Elaine, Age 42, 4 Y.o.E)*

Yet, establishing a clear project structure requires a thorough consideration of the organizational and structural differences of partners and their organizations, as well as communicating the structure to all relevant stakeholders. For example, a respondent mentioned that he had to understand the differences in partnering organizations' funding logics and clarify internally (within the leader's organization) and externally (to their partnering organizations) how the new funding structure worked:

“*… we had to clarify internally, but it also had to be clarified with [the partnering institutes]… This was also an unfamiliar approach for them because other funding logics simply work differently than health insurance funding, both in science and in sports.” (Moses, 56, 2 Y.o.E)*

Meanwhile, early team-building measures were vital for enabling diverse partner members to get to know each other better on a personal level even before the project started officially. Although such activities can be highly time-consuming and costly, interviewees found them helpful in reconciling partner members' perspectives and working styles, which later improved their project involvement:

“*That was quite a lot of effort, time-consuming for all people. But what I found interesting was that everybody was involved... you get to know each other… I found it very helpful at the time because it loosened up the atmosphere a bit… you got to know people beyond their professional competence.” (Bonnie, Age 35, 2 Y.o.E)*

Therefore, many project leaders saw the need to invest time and effort in planning, establishing clear project structures, formulating goals with partners and engaging in team-building activities *as early as possible*. These activities could help partner members align their interests and resources, establish better personal relationships, and lead to smoother project functioning later on.

### Strategy 2: Inclusive and transparent decision-making (throughout the project)

While interviewees often relied on their foresight, intuition, or feelings to determine when to engage partners in major decisions or how to communicate with them; they also recognized the need to be decisive in making decisions to ensure project progress, especially for larger projects that involve multiple partners:

“*When you have so many partners, you naturally want to make decisions together… however, it is still important for a project manager to be able to make decisions… If it comes to the fact that there are problems… you have to hit the table and decide.” (Elaine, Age 42, 4 Y.o.E)*

A project leader also highlighted that it was critical to establish a framework and safe space for community partners to enable a highly inclusive decision-making process: “*You have to be very close [to the community partners] and provide a framework so that a “safe space” is created. They [The community partners] bring a lot of resources with them, but we [leadership team] have to set the framework.” (Iris, Age 35, 2 Y.o.E)*

Although not all decisions were jointly made, interviewees underscored the necessity to involve partners in discussions early on and ensure a transparent decision-making process *during the project*. This could be achieved by ensuring proper documentation (e.g., minutes or summary reports), which ensures the transparency of all decisions and agreements. One of the participants noted: “*After each meeting, everyone has a different understanding of what was discussed, to put it exaggeratedly. And such minutes help us immensely to make progress and agree on the next steps based on the joint minutes.” (Marie, Age 36, 2 Y.o.E)*

Similarly, keeping a daily project diary throughout the project helped a project leader stay aligned with prior decisions and directions, which was a key determinant for project quality and success: “*We keep a project diary in every project, where we write something down every day…That is a crucial success factor. By the way, it's also a quality factor. Otherwise, you do something else after half a year.” (Walter, Age 56, 6 Y.o.E)*

Accordingly, proper documentation is vital to keep the decision-making process inclusive and transparent. It also helps project partners to build on prior agreements and decisions and clear up any misunderstandings, thus accelerating the project's progress and promoting its quality and success.

### Strategy 3: Interdisciplinary-friendly environments (throughout the project)

A few project leaders underscored the necessity of ensuring an interdisciplinary-friendly environment for partners *throughout the project*. For instance, they would master the skills, knowledge, or perspectives from other disciplines; and foster networking and lateral thinking skills, which, according to one of them, is the ability to “*link things that are not really connected”*: “*Everyone has different aspects, even from their training, which they bring to the team. And this networking and lateral thinking result in teamwork.” (Elaine, Age 42, 4 Y.o.E)*

Other interviewees proposed strategies to ensure clear and comprehensible communication for interdisciplinary partners, such as by creating a glossary to clarify any technical terms in each meeting or by involving interdisciplinary specialists to establish an effective communication structure *from the start of the project*:

“*Right at the beginning... we decided that we would get support and hired two people from a university who know about interdisciplinary work. They have always come to our meetings and listened, for example, how do we communicate? How is that received by everyone?... which worked quite well.” (Bonnie, Age 35, 2 Y.o.E)*

Moreover, leaders who led team members in the absence of disciplinary hierarchy often could not direct or decide partners' pace of work in a networked project. A useful strategy was to ask for project updates regularly, to detect any challenges, and to persuade partners to make progress *during project implementation*. Cultivating a strong personal connection between partners also assisted them in overcoming communication problems and promoted effective collaboration. One of the interviewees underlined: “*At the beginning... there have been some misunderstandings and communication problems. But in the end, I think we have come to terms with each other and got to know each other so well that it went pretty well.” (Max, Age 68, 14 Y.o.E)*

Creating a friendly project environment on both personal and professional levels was crucial to overcome differences across disciplines and facilitate effective ongoing communication.

### Strategy 4: Responses to project changes and uncertainties (throughout the project)

To handle unexpected project changes that arise during the project implementation, a few interviewees highlighted the importance of being approachable for questions, discussions, and prompt clarification: “*I am approachable - always, at all times in the project.” (Nelson, 46, 14 Y.o.E)*

Meanwhile, project leaders' experience significantly influenced their adaptability, resilience, and patience in responding to dynamic project environments. For example, more experienced project leaders explained that they acquired the capability of anticipating changes over time, thus were more comfortable in improvising or finding detours upon changing project situations:

“*Experience also does something to you, that you simply know there is nothing that runs smoothly and everyone who has ever done a project knows that no project is ever implemented the way it was created. Something always happens (laugh). Yes, and in this respect, you need a bit of flexibility and at the same time... you always have to know: ‘where are we going?”’ (Annie, Age 45, 5 Y.o.E)*

Thus, being available for others, anticipating changes and remaining flexible *throughout the project* were essential for effectively adapting to unforeseen project circumstances.

### Strategy 5: Tactical project development and implementation (post-project)

In response to the challenges of lacking project impact and sustainability, a few respondents noted the necessity to consider and explore any opportunities to continue their endeavor *at the end of the projects*. Apart from applying for follow-up funding, one way to ensure project impact and sustainability was to develop a strategic research agenda to retain staff and conduct more projects in the same area:

“*You have to acquire a strategy… That means: how do you promote this [research topic] over the years? And they have to converge thematically…so that (a) I can handle it with my team of people and (b) they stay with me so that I can pursue my research line?” (Walter, Age 56, 6 Y.o.E)*

Sometimes, that also implies ensuring the project's strategic orientation fit the different interests of relevant parties. For example, a respondent noted:

“*In terms of content, for me it is a matter of ensuring that the strategic orientation of this project.... This means that I have to keep my entire health reporting [of the city] in mind… but I also have to keep an eye on the strategic orientation of urban renewal. There are overlaps, but they also have their own interests in this.” (Moses, Age 56, 2 Y.o.E)*

Alternatively, one could transfer the project idea to other contexts or work pragmatically with existing resources and capacities to ensure project quality and impacts:

“*We always work within a framework and with the resources available to us, so as not to overburden anyone or anything; because that always leads to measures being implemented inadequately or unsatisfactorily. That's why I think, and here I believe in a more sustainable sense, that I look at ‘what's there’ and try to implement the project objectives.” (Jasmine, Age 35, 2 Y.o.E)*

Hence, strategically planning for the research agenda and transferring project results based on existing resources and outcomes contributed to maintaining a project's impact and sustainability beyond the project cycle.

### Effective leader qualities

Together with effective strategies, we also identified four qualities that leaders should possess to effectively lead in CAHP projects. They included adopting a proactive attitude, having an open and adaptive mindset, relying on peer learning and support, and emphasizing self-growth and reflexivity.

### Quality 1: Proactive attitude

Whilst many CAHP project leaders explained that they have a coordinating or enabling role in the projects, a few interviewees emphasized the significance of being proactive in asking for new information to understand the project content or to shape changes to make progress in their projects: “*You have to be flexible, trust yourself; but at the same time, be active... you have to be willing to shape changes.” (Olivia, Age 29, 3 Y.o.E)*

Sometimes, it also implies that they must set an example to motivate partner members to engage in the project or to rise to any challenges proactively: “*I have to be a role model. I have to do more, know more and always want to… I have to rise to the challenges… If I'd rather not put so much effort into it, then it won't work.” (Walter, Age 56, 6 Y.o.E)*

Project leaders can also actively involve policymakers or the press to promote their projects' vision, visibility, and acceptance. For example, an interviewee working on a highly controversial health topic has noted the significance of lobbying and media work on his project: “*We were called names there. We had a television crew every week… We were in every major national newspaper… Public opinion was absolutely on our side… So, we work intensively with the media.” (Walter, Age 56, 6 Y.o.E)*. Over the years, the project has become one of the successful model projects that convinced former opponents to cooperate and drove several legal changes at the federal level.

Therefore, besides enhancing project-level engagement, leaders' proactivity in advocating for their projects could also radiate to a societal level. This could lead to more significant project impacts and external support from the project environment or society.

### Quality 2: Open and adaptive mindset

Despite many project leaders mentioning the need for project planning in advance, each project can be highly different and susceptible to uncertainties. Therefore, it is vital for project leaders to adopt an open and adaptive mindset, to keep an ear open for feedback and criticism and to adjust their leadership styles constantly:

“*We don't get much feedback from colleagues at my level now...you don't really get much feedback as a leader… However, if they don't react to me, I have no idea how to put it… And vice versa, giving feedback [to others]. Even if it's critical [feedback], stand by it. Otherwise, we won't get anywhere together.” (Walter, Age 56, 6 Y.o.E)*

More experienced project leaders also learned to improvise and accept that some things cannot be controlled directly. Instead, they had to be constantly prepared for new challenges and be able to identify and take alternative paths to achieve the same goal when contingencies occurred. One of the participants pointed out:

“*You certainly have a rough goal and a direction in mind, but you have to be prepared to deviate from the seemingly emerging path under certain circumstances and to take a better path instead, and I think it is important to try to maintain this openness and also to communicate it.” (Barry, Age 64, 9 Y.o.E)*

Thus, an open and adaptive mindset allowed leaders to redirect their measures to meet their project goals readily.

### Quality 3: Peer learning and support

When making major decisions on complicated issues beyond their scope of expertise, many project leaders would actively discuss or seek advice and support from peers, including their network/partner members, colleagues, experts, or superiors from their organizations: “*Most things are not decided alone but always, at least with my closer team or with the methodological director of the project, who works in [city name] at the university. I discuss this with him.” (Claire, Age 40, 6 Y.o.E)*

Alternatively, when there is an absence of role models to refer to in an innovative project, project leaders note that a good way to cope is by reaching out to external experts to learn from their experiences. For instance, one respondent mentioned:

“*Unfortunately, we did not have so many role models. That means that next time I would perhaps try to network more, also outside the [affiliated organization]… I would probably get help directly from others, perhaps other funds or projects, and simply conduct an interview (laughs) and ask: ‘What have your learnings been? And what can you recommend to me?”’ (Marie, Age 36, 2 Y.o.E)*

Meanwhile, other interviewees expressed the benefits of having supportive staff or complementary colleagues in assisting project implementation: “*But realistically, I think the key is to have the right people to support you. So, I'm in the fascinating and amazing position that I have great people whom I can count on.” (Natalie, Age 45, 10 Y.o.E)*.

Therefore, peer exchanges and support enabled project leaders to identify ways to deal with complex, challenging, or critical situations and implement their projects more effectively.

### Quality 4: Self-growth and reflexivity

Several project leaders reflected on the importance of self-growth and reflexivity in leadership practices. These enabled them to perform more effectively in (future) CAHP projects, such as building on previous leadership experiences and being prepared to learn new things constantly:

“*When you are that old, you can build on your experience, and you are constantly learning. And I think that was an important asset for me… The best example to prove that you can do it is that you have done it before, successfully. And I think that's how it works in many areas in life and also here in this particular field of science.” (Barry, Age 64, 9 Y.o.E)*

That learning process includes taking part in management training or learning-by-doing. In addition, understanding one's leadership styles, strengths, and weaknesses remains critical for improving the ability to lead CAHP projects effectively. Such reflexivity in leadership practices and self-criticism helped project leaders think about their self-image, reflect on their role models, and summarize their learnings. For instance, a respondent noted:

“*Being able to look back, why is it now? Is that so now? I believe that this is a crucial variable: the ability to reflect… I have to reflect on it, and I have to restructure everything somehow. This ability to reflect and then open up; instead of standing still and burying our heads in the sand, look at it and deal with it openly.” (Nelson, Age 46, 14 Y.o.E)*

Hence, the conscious, continuous cycles of self-reflection helped leaders restructure their leading experience and improve their ability to lead more effectively.

Based on the above findings, we constructed a process model summarizing how effective CAHP project leadership and leading can be achieved (see, Figure 1).

## Discussion

Although prior CAHP and CLT research has highlighted the influential role of effective project leadership in driving successful partnership outcomes (27, 37), *how* this is achieved in different CAHP settings remains under-defined and under-researched (15, 18). Therefore, through an interpretivist approach, this study purposively approached project leaders of various CAHPs in Germany to explore their perspectives on effective leadership and leading in their unique project settings.

Our findings reveal several insights into the meaning of effective *leadership* and effective *leaders* and suggest the dynamic strategies, qualities, logics, and processes needed to enhance effective CAHP project execution by juxtaposing CLT's operational, enabling, and entrepreneurial leadership logics (Figure 1).

### Effective leadership strategies in CAHP projects

Our findings suggest that project leaders may face similar leadership challenges within a CAHP project cycle. Despite the differences in project team composition, project size, and thematic foci, these challenges (i.e., project planning and management, the balance of participatory decision-making, project interdisciplinarity, project changes and uncertainties, and lacking project impacts and sustainability) are known in the CAHP literature (4, 11, 13). Besides corroborating these challenges, our study further highlights the effective strategies that facilitate project leaders in nonhierarchical, complex CAHP settings to perform their *leadership* tasks effectively. Our findings also indicate that these strategies, functioning as dynamic responses to emergent challenges, align with the operational leadership logic of the triadic complexity leadership model (25). For instance, project leaders displayed operational leadership behaviors (i.e., structuring tasks, resources, roles, and responsibilities) while tackling project planning and structural issues. They also actively coordinated with partners and created the inclusive, transparent, and interdisciplinary-friendly environments necessary for participatory decision-making and meaningful collaboration while dealing with decision-making and interdisciplinary communication challenges.

In addition, our findings extend the literature on effective CAHP functioning (10, 29, 38) by unraveling how these strategies promote smooth CAHP project operations by reinforcing facilitating factors of effective collaboration (i.e., project inputs and resources, roles and procedures, communication). Our evidence shows that careful project planning and management can secure adequate inputs and resources for project task implementation. Similarly, participatory decision-making and project efficiency can be reinforced by establishing a clear decision-making structure and delineating partners' roles and responsibilities after understanding partners' unique structural needs. Likewise, effective communication can be strengthened *via* fostering lateral thinking, creating interdisciplinary-friendly environments, or channeling information based on partners' engagement levels.

### Effective leader qualities in CAHP projects

Secondly, our study contributes to the theoretical advancements of leadership development in complex adaptive network settings by pointing to a leader's active learning-oriented, individual growth process. Our empirical evidence echoes literature on the enabling leadership logic of CLT (22), suggesting that CAHP project leaders often had an enabling role on top of an operational one. They also found themselves most effective in performing their roles when they actively customized their leadership approaches according to their structural and relational dynamics with project partners, instead of adopting specific leadership “styles”. Meanwhile, extant literature generally assumes that a project leader's ability to excel in CAHP projects depends on their professional judgement built upon leadership experiences (2, 39). However, given the heterogeneity, complexity, and uniqueness of each CAHP project (37), project leaders (particularly those lacking such background knowledge and experiences) can only identify the most effective approaches by constantly experimenting and renewing their learnings in a collaboration process (26). Our findings show that four qualities enable CAHP project leaders to lead more effectively, namely: (1) adopting a proactive attitude to move projects forward; (2) having an open and adaptive mindset to embrace learning and leadership improvement opportunities; (3) relying on peer learning and support in addressing leadership challenges; and (4) emphasizing self-growth and reflexivity to improve leadership practices continually. These findings resonate with Bucknall et al.'s (2021) proposition that CAHP project leaders perform better if they remain approachable, are open to conversations and ideas, and are willing to learn and explore new research areas. In line with the proposition of complexity leadership that leaders nowadays must be more flexible, agile, and adaptive in an ever-changing and unpredictable world (25), our findings further elaborate on *how* leaders' deliberate efforts in active learning can help them lead better in complex, ambiguous and heterogeneous CAHP project environments. For example, project leaders' proactive attitudes in shaping changes or rising to challenges help them establish the credibility and legitimacy required to make progress in nonhierarchical, shared power arrangements like CAHPs. As such projects often involve multi-stakeholder effort in innovation and cocreation (31), project leaders' abilities to constantly learn, adapt to new environments and seek support from peers facilitate them to identify innovative approaches for solving community health issues. Thus, our findings indicate that effective project leaders must acquire a growth mindset to strengthen their proactivity, openness, adaptability, resourcefulness, and self-growth in a CAHP project cycle.

### The dynamic developmental logics and processes of effective CAHP project leaders(hip)

Thirdly, given that extant CLT literature primarily focuses on complex network interactions instead of positional leaders' contributions (28), our research extends the CLT literature by accounting for the differences between effective *leadership* and effective *leaders* in complex, networked project settings like CAHPs (23). Our research also illustrates the contributions of their developmental logics and processes to enhanced project functioning in a CAHP project cycle. Unlike the linear entrepreneurial-enabling-operational leadership emergence sequence proposed by Uhl-Bien et al. (25), our findings suggest that effective CAHP project leadership emerges from dynamic, fluid changes between the three forms of complexity leadership logics throughout the project cycle. Even though the entrepreneurial leadership logic can be seen as the primary force initiating and driving the cycle, it requires the project process to adapt to the changing or uncertain environments constantly. Hence, only in combination with the other logics can the entrepreneurial process effectively move forward until new opportunities need to be identified for future projects to address the challenge of lacking project continuity and sustainability. Each leadership logic (operational, enabling, and entrepreneurial) thus allows CAHP project leaders to accomplish their versatile leadership tasks concerning project operation, partner relations, and project uncertainty.

Together, CAHP leaders' ability to use the three logics flexibly and in situationally-appropriate ways enhanced the overall project functioning and prevented major subsequent leadership challenges. For instance, adopting an *operational leadership* logic *during project implementation* can help project leaders to create structures, resources, and routines necessary for smooth operation and high project performance and efficiency. Meanwhile, *enabling leadership* logic was crucial for sustaining partner relations and effective leading *throughout the CAHP cycle*. Creating interdisciplinary-friendly environments and fostering relationship-building among partners were essential for establishing trustful personal bonds and resolving subsequent tensions, conflicts, and miscommunication. On the other hand, in the face of persistent project uncertainty (particularly at *pre-and post-project phases*), project leaders may perform their leadership roles more effectively by adopting an *entrepreneurial leadership logic*. This logic allows them to proactively explore and ideate new project opportunities, experiment with novel solutions, or generate paths for sustainable project development. Thus, our findings suggest that project leaders must act under various leadership logics to meet the CAHP's needs for project performance and meaningful knowledge cocreation to develop effective leadership in interorganizational, networked CAHP project settings.

We also found that leaders' identities in CAHP projects could be unstable or evolving, as suggested by Tourish et al. (23). Hence, for CAHP leaders to lead their projects effectively, they should constantly build on the four identified qualities (i.e., being proactive, adaptive, resourceful, and self-growing) *throughout the project cycle* and repeat the same learning cycle in each CAHP project. Reinforcing these qualities would help them develop and evolve into effective leaders over time and strengthen their ability, readiness, and legitimacy to lead as enablers in nonhierarchical and ever-changing CAHP settings. Our proposed process model (Figure 1) provides a unifying theoretical account of the organic task execution and qualities required for CAHP project leaders to achieve high leadership effectiveness. The model highlights the iterative cycle of how project leaders may continuously learn, adapt, evolve, synthesize, and transfer their learnings into their leading process to effectively fulfill their leadership functions and leader roles in new (CAHP) project environments.

### Practical implications

Whilst previous studies have investigated effective leadership at a specific project stage (i.e., formation and ending phases) (7, 21), our study captures a full spectrum of empirical insights into effective leading throughout the project cycle by examining CAHP projects in different stages. We also differentiated between effective leadership and effective leaders to synthesize the components of effective leading from diverse CAHP projects, ranging from newly formed to successful follow-up partnerships and those of varied complexity, power dynamics, and sizes. In so doing, our proposed model offers practitioners in CAHP project leadership roles a framework to translate effective leadership into practice. More specifically, the framework provides clear directions on what project leaders can do to prevent and/or navigate the challenges they may face in implementing CAHPs (17).

Another important practical implication from our findings is that although project leaders may address the leadership challenges differently (40), the overarching process through which they can lead effectively can be similar (25). For instance, project leaders can be operational by establishing a clear structure or routine for project practicalities like efficiency and performance. Within the predefined project structure and routine, they may create a flexible and adaptive space or culture to enable innovation and cocreation while embracing the unique tensions, ambiguity, and uncertainty. They may also be entrepreneurial in seeking new ways and plans to adapt to changing environments in a dynamic project process. Thus, the leading process illustrated in our model can offer project leaders a visual synopsis of the fundamental steps to ensure effective CAHP leading.

Moreover, although researchers are often automatically assigned a leadership role to manage CAHP projects (37), our findings indicate that some might not be fully trained or mentally prepared to take up such positions, thus resulting in the risk of indecisiveness and mismanagement due to inexperience. Therefore, our study echoes previous literature (25, 30, 31) by demonstrating the necessity for CAHP project leaders to reinforce their cognitive skills and resilience in handling the project complexity through leadership training. Our evidence also supports (19) that an alternative for project leaders lacking leadership training or support from their affiliated organizations is to leverage their personal (cognitive) resourcefulness. For instance, apart from learning-by-doing the tasks necessary for effective leadership, they may also proactively sustain or boost the project momentum, possess an open, adaptive mindset to handle any project contingencies, and actively seek advice and support from their partnership networks, experts, colleagues, or peers. To become better leaders, project leaders should also develop a growth mindset (30) and be open to new ideas and critical feedback from others.

Our evidence suggests that this cognitive, growth-oriented quality is especially relevant for experienced and high-status project leaders since they may not receive as much feedback on executing their leadership as their inexperienced junior counterparts, thus failing to sense any issues or room for improvement. Therefore, we suggest that CAHP project leaders should regularly engage in open discussions with their peers or partner members in learning communities to share practices and gain critical feedback. Regardless of their experiences and status in the affiliated organizations, they should continuously reflect on their leadership tasks and behavioral qualities in recent practices to improve their leadership effectiveness in complex and constantly evolving CAHP settings. Alternatively, we recommend that experienced CAHP project leaders actively provide and promote leadership training, mentoring, and/or coaching to their successors or peers. This ensures that the extensive practice and hands-on experience, together with the valuable tacit knowledge accumulated over time, are not dissipated and can be passed on as they retire or change positions.

### Limitations and future research implications

As with all research, this study is also subjected to limitations. Firstly, readers should remember that our new model discusses how project leaders can perform their leadership functions and roles effectively through different strategies and develop themselves into effective leaders in unique CAHP settings. Hence, the leadership strategies and qualities can be limited to positional leaders' perspectives. We tried to reduce this bias by asking project leaders how major decisions were made in the project instead of their leadership styles, and also by asking them to support the ways of leading they described with concrete behavioral examples. However, from a CLT perspective, leadership is not confined only to positional leaders (25). Effective leadership can also be coconstructed by interacting individuals (27). Indeed, a growing body of literature has highlighted the potential for developing collective and shared leadership capacity (39) and mutual/collaborative learning skills in a partnership (6, 20). Thus, project partners' leadership skills and qualities may also significantly augment effective CAHP project implementation. Whether partner members should possess the same qualities as project leaders and their potential synergetic effect at the project level deserve further research. Future research may explore the applicability of the proposed strategies and qualities to project partners (who are not in formal project leadership positions) or to the collective level. Researchers may also validate the model by conducting an ethnographic or longitudinal observational study on carefully nominated, effective CAHP leaders to examine if the proposed strategies and qualities are reflected.

Secondly, although our research covers a broad perspective of leaders from diverse CAHP projects, our study is based on a heterogeneous purposive sampling (pp. 337) (32) and is limited to projects specific to the German context. Thus, it may have limited generalizability due to its nonprobability sampling and cultural embedding (pp. 296) (32). Therefore, our findings should be interpreted cautiously. Yet, Germany is well known for its capability to organize. Thus, studying and reflecting on German project leaders' experiences may not be so limiting after all. Also, it is worth mentioning that German projects financially supported by the ministries or private nonprofit foundations often strongly align with the German welfare regime (37). Indeed, most CAHP projects reported in this study were third-party funded projects formally led by academic researchers. Therefore, the leadership challenges and strategies reported here might be more specific to academic leaders and are tinged with research-oriented and power imbalance issues (38). Future studies should explore the transferability of our model to other contexts, such as other interdisciplinary projects, or bottom-up, grassroots CAHP projects initiated or led by community stakeholders, where the power dynamics and project structures may differ (16). Thirdly, although the transcripts were sent to interviewees for checking to ensure their accuracy, we did not perform member checking by sharing the completed analysis with interviewees. Our decision was based on Morse's argument (2015), according to which this strategy was not recommended due to its limited value in attaining validity and reliability and the potential negative impact on analysis objectivity (41). However, we followed Morse's suggestion to enhance the credibility of our findings by checking for the presence of any normative behavioral patterns among CAHP project leaders during concurrent data collection and analysis. We did so by referring to other participants' comments during data collection, asking the following question: “Other interviewee(s) mentioned [a specific situation or a response to the same or similar situation]. What was it like in your situation?” (41). Future studies could consider using this approach to further improve credibility of findings.

## Conclusion

This study examines the leadership dynamics within the complex realities of CAHPs by underlining the significant yet poorly understood role of project leaders in CAHP project orchestration. Our work links state of the art complexity leadership, wicked problems, and leaders(hip) development processes to illustrate how project leaders in diverse CAHP settings can effectively operate. We differentiated effective leadership from effective leaders and unraveled the strategies, qualities, logics, and processes that support CAHP project leaders to enact leadership and lead more effectively. Extra attention should be dedicated to the selection, development, and monitoring of project leaders' leadership effectiveness and their preparedness in leading CAHPs to ensure fruitful coconstruction between diverse academic and community partners and to fulfill their promise of bringing long-term health benefits to the members of the targeted populations.

## Data availability statement

The original contributions presented in the study are included in the article/Supplementary material, further inquiries can be directed to the corresponding author/s.

## Ethics statement

The studies involving human participants were reviewed and approved by the BMS Ethics Committee, University of Twente. All participants provided their written informed consent to participate in this study.

## Author contributions

CC led the data collection and analysis. Both authors contributed to the writing and revision of the manuscript and approved the final version of the submitted manuscript. Both authors have made a substantial, direct and intellectual contribution to the study conception and design and data interpretation.

## Funding

CC was supported by a grant (03IHS062A) from Innovative Hochschule, a joint initiative by the Bundesministeriums für Bildung und Forschung (German Federal Ministry of Education and Research) and the Gemeinsame Wissenschaftskonferenz (The Joint Science Conference of the German Federal Government). These funders had no further role in the study's design, data collection, analysis, or writing of this publication.

## Conflict of interest

The authors declare that the research was conducted in the absence of any commercial or financial relationships that could be construed as a potential conflict of interest.

## Publisher's note

All claims expressed in this article are solely those of the authors and do not necessarily represent those of their affiliated organizations, or those of the publisher, the editors and the reviewers. Any product that may be evaluated in this article, or claim that may be made by its manufacturer, is not guaranteed or endorsed by the publisher.
