# Supplementary material for: Effective leaders(hip) in community-academic health partnership projects: An inductive, qualitative study
Source: Front Public Health. 2022 Aug 12;10:941242. doi: 10.3389/fpubh.2022.941242 (PMC9411517; doi:10.3389/fpubh.2022.941242)
Supplement: Supplementary file 1 [file Data_Sheet_1.DOCX]

Supplementary Material

**Supplementary Material 1. Participant Overview (*N* = 32)**

| **Identifier** | **Pseudonym** | **Age** | **Gender** | **Affiliated position (type of affiliation)^1^** | **Project partners^1^** | **Years of experience in project leadership** | **Project duration** | **Number of project years** | **Project theme** | **Funding source^2^** |
| --- | --- | --- | --- | --- | --- | --- | --- | --- | --- | --- |
| **P01** | Anna | 53 | Woman | Professor (U) | E, HCI, U | 12 | 2015 - 2020 | 6 | Education and training for health professionals | B |
| **P02** | Bonnie | 35 | Woman | Researcher (R) | H, R | 2 | 2013 - 2020+ | 8 | Disease prevention | FS, M |
| **P03** | Claire | 40 | Woman | Professor (U) | HCI, P, R | 6 | 2018 - 2020 | 3 | Treatment/Care improvement | S |
| **P04** | Daisy | 46 | Woman | Post-doc researcher (U) | C, HCP | 4 | 2019 - 2021 | 3 | Treatment/Care improvement | FS |
| **P05** | Elaine | 42 | Woman | Manager (C) | C, H, U, NGO | 4 | 2016 - 2020+ | 5 | Treatment/Care improvement | EU |
| **P06** | Gary | 40 | Man | Chief executive officer & founder (C) | C, R, U | 5 | 2015 - 2020+ | 6 | Treatment/Care improvement | FS |
| **P07** | Helen | 50 | Woman | Private lecturer (U) | P, R | 10 | 2017 - 2020+ | 4 | Disease management | FS |
| **P08** | Iris | 35 | Woman | Officer (NGO); researcher (U) | CS, HP | 2 | 2019 - 2020+ | 2 | Community health promotion | I, S |
| **P09** | Jasmine | 35 | Woman | Research associate and lecturer (U) | CS, G, NGO | 2 | 2018 - 2020+ | 3 | Community health promotion | S |
| **P10** | Kelly | 50 | Woman | Worker (NGO) | C, G, HCI | 4 | 2020 - 2021 | 2 | Education and training for health professionals | I, S |
| **P11** | Lily | 34 | Woman | Coordinator (H) | CS, H, HCI, R, U | 2 | 2019 - 2022 | 4 | Disease management | FS |
| **P12** | Max | 68 | Man | Senior professor (U) | C, HCI, U | 20 | 2015- 2020 | 6 | Community health promotion | FS |
| **P13** | Nelson | 46 | Man | Professor (U) | G, HCP, NGO, U | 14 | 2019 - 2022 | 4 | Community health promotion | FS |
| **P14** | Olivia | 29 | Woman | Deputy commercial director (H) | C, HCI, U | 3 | 2017 - 2020+ | 4 | Treatment/Care improvement | P |
| **P15** | Paul | 55 | Man | Professor (U) | G, HCP | 14 | 2020 - 2022 | 3 | Education and training for health professionals | EU |
| **P16** | Queenie | 64 | Woman | Project leader (H) | C, CS, U | 22 | 2018 - 2020+ | 3 | Patient support | P |
| **P17** | Ron | 26 | Man | Research associate (U) | G | 2 | 2018 - 2022 | 5 | Health promotion | S |
| **P18** | Sophie | 54 | Woman | Department head (G) | G, HCI, NGO, U | 9 | 2019 - 2020+ | 2 | Patient support | FS |
| **P19** | Tina | 55 | Woman | Staff (G) | G, HCP, U | 5 | 2015 - 2021 | 7 | Community health promotion | S, I |
| **P20** | Ulva | 55 | Woman | Advisory board member (NGO); freelance lecturer (E) | G, NGO | 10 | 2019 - 2020+ | 2 | Patient support | P |
| **P21** | Victor | 62 | Man | Professor (U) | C, HCI, NGO | 6 | 2018 - 2020+ | 3 | Treatment/Care improvement | S |
| **P22** | Morton | 45 | Man | Professor (U) | C, H, U | 4 | 2016 - 2019 | 4 | Treatment/Care improvement | FS |
| **P23** | Karen | 30 | Woman | Manager (H) | H, HCI, HCP, R | 0 | 2020 | 1 | Treatment/Care improvement | FS |
| **P24** | Carla | 49 | Woman | Professor (U) | CS, G, HCP | 5 | 2015 - 2020 | 6 | Community health promotion | S |
| **P25** | Barry | 64 | Man | Professor (U) | I, H, HCP, U | 9 | 2020 - 2022 | 3 | Treatment/Care improvement | FS |
| **P26** | Walter | 58 | Man | Managing director (R) | HCI, U | 6 | 2017 - 2020 | 4 | Treatment/Care improvement | FS |
| **P27** | Annie | 45 | Woman | Professor (U) | CS, NGO, U | 5 | 2018 - 2021 | 4 | Treatment/Care improvement | S |
| **P28** | Marie | 36 | Woman | Management officer (I) | I, NGO, U | 0 | 2019 - 2023 | 5 | Treatment/Care improvement | FS |
| **P29** | Moses | 56 | Man | Managing director (G); health reporter | CS, G, NGO, U | 0 | 2019 - 2021+ | 3 | Community health promotion | I |
| **P30** | Janet | 31 | Woman | Project manager (U) | C, H, HCI, G, I, U | 3 | 2018 - 2022 | 5 | Community health promotion | S |
| **P31** | David | 42 | Man | Research associate (U) | E, HCP, NGO, U | 3 | 2018 -2021 | 4 | Community health promotion | S |
| **P32** | Natalie | 45 | Woman | Professor (U) | C, H, NGO, I, U | 10 | 2020 - 2022 | 3 | Community health promotion | I |

Notes: ^1^ U= Universities; E = Educational institutions (e.g., schools, training centers); HCI = Health care institutions (e.g., clinics, health care centers, private practices); R = Research institutes; P = Patients; HCP = Healthcare professionals (e.g., doctors, nurses); H= Hospitals; C = Companies; CS = Citizens; NGO = Nongovernmental organizations (e.g., nonprofit organizations, charities); G = Government authorities/policymakers; I = Insurance companies

^2^ B = Bank; FS = Federal state funding; M = Membership fee; S = State funding; EU = European funding; I = Insurance company; P = Private funding

**Supplementary Material 2. Final Interview Protocol**

| Introduction and purpose (5 minutes) | | |
| --- | --- | --- |
| Greetings  Introduction | - Welcome and self-introduction (Name, Affiliation) - Research background - Aim: To find out what challenges CAHP project leaders encounter and how they deal with those challenges to perform well. | - Begrüßung und eigene Vorstellung (Name, Zugehörigkeit) - Grund der Untersuchung - Ziel: Um die Herausforderungen die CAHP-Projektsleiter*innen konfrontiert und wie sie mit diesen Herausforderungen umgehen und gute Leistungen erbringen herauszufinden. |
| Interview structure | This interview will be structured as follows:   - First, we will ask some general questions about you and your partnership project. - Afterwards, we will talk about the leadership in this project, the influences of any (major) challenges on your project, and how your project team (and you as a project leader) reacted to them. - Finally, we will discuss the key factors that impact your project team’s performance. - Duration: 30-60 minutes | Struktur des Interviews erwähnen:   - Zunächst werden wir einige allgemeine Fragen zu Ihnen und Ihrem Partnerschaftsprojekt stellen. - Anschließend sprechen wir über die Führung in diesem Projekt, die Einflüsse von (großen) Herausforderungen auf Ihr Projekt und wie Ihr Projektteams (und Sie als Projektleiter*in) auf diese Ereignisse reagiert haben. - Zum Schluss werden wir die Faktoren besprechen, die die Leistung Ihrer Projektteams beeinflussen - Geplante Dauer (ca. 30-60 Minuten) |
| General Data Protection Regulation (GDPR)  Confidentiality  Anonymity | Before we start:   - Declaration of consent sent in advance (received and signed) - Explain GDPR orally and obtain permission: This conversation is strictly confidential and content recorded will not leave this meeting and therefore will not be passed on to third parties. - All data collected will only be used for research purposes, and quotes might be mentioned in scientific reports anonymously. | Bevor wir anfangen:   - Einwilligungserklärung vorab zugeschickt (und signiert erhalten) - DSGVO mündlich erklären & Erlaubnis einholen: Dieses Gespräch ist streng vertraulich und wird diesen Raum nicht verlassen und somit auch nicht an Dritte weitergeleitet werden. - Alle gesammelten Daten werden nur zu Forschungszwecken verwendet und Zitate können in wissenschaftlichen Berichten anonymisiert erwähnt warden. |
| Consent & Recording | - Do you have any questions? - Ask for permission to record conversation. - Start recording. | - Haben Sie Fragen? - Aufzeichnung des Gespräches erwähnen und Einverständnis einholen. - Tonaufnahme starten. |
| General questions (5-10 minutes) | |  |
| Introduction of the interviewee | - Age - Job - Years of experience in leading/managing CAHP projects. - For how long have you been working on this project? | - Alter - Beruf - Wie viele Jahre Erfahrung im sektorübergreifende Projektsleitung/-management zwischen Forschern und gesellschaftlichen Akteuren. - Beschäftigungsdauer im Projekt |
| Project details  Partnership structure | - Could you please briefly describe the project? - Project duration - Formal project goals at the start of the project - Project partners: With whom are you currently working on this partnership project? - Can you briefly explain how this collaboration with the partners works? (e.g., to what extent is each partner involved in the project?) - Can you describe which phase your project is currently in? (e.g., Initiation /Planning/Implementation/Monitoring/Closing phase) | - Wie würden Sie kurz das Projekt mit Ihren eigenen Worten beschreiben? - Dauer des Projektes - Formale Projektziele zu Beginn des Projekts - Projektspartners: Mit wem arbeiten Sie im Rahmen des Projektes zusammen? - Können Sie kurz erklären wie diese Zusammenarbeit mit den Partnern funktioniert? (z. B. Inwieweit ist jeder Partner in das Projekt eingebunden?) - Können Sie beschreiben, in welcher Phase sich Ihr Projekt gerade befindet? (z. B. Initiierungs-/Planungs-/Aktions-/Monitoring-/Abschlussphase) |
| Questions about leadership (5-10 minutes) | |  |
| Leadership and decision-making processes | - How did you become a leader of this project? - What are your tasks as project leader/manager? /How would you describe your role in the project? - How are major decisions made in the project?   - Why? - How would you describe the ways of leading the project?   - How does that work in practice?   - Can you give me some examples to illustrate your answers? | - Wie sind Sie in diesem Projekt zum Leiter*in geworden? - Aufgaben als Projektleiter*in/Manager*in/ Wie würden Sie Ihre Rolle in diesem Projekt beschreiben? - - Wie werden wichtige Entscheidungen im Projekt getroffen? - Wieso? - Wie würden Sie die Art und Weise beschreiben, wie Sie das Projekt leiten?   - Wie funktioniert das in der Praxis?   - nach einige Beispiele fragen |
| Questions about challenges, enablers, and performance (10-15 minutes) | | |
| Status quo/ performance | - How is your project going at the moment? - Any achievements? - Any major hindrances/challenges? - Did the predefined goals change or have they been adapted in the course of the project? - If so, how? - Why were they changed? - How would you rate the project team’s performance in achieving the intended project goals? (Very Low 1-5 Very High) - Why? - What can be improved? | - Wie läuft Ihr Projekt im Moment? - Gibt es Erfolge? - Gibt es größere Hindernisse/Herausforderungen? - Haben sich die vordefinierten Ziele im Laufe des Projekts geändert oder angepasst? - Wenn ja, wie? - Warum wurden sie geändert? - Wie beurteilen Sie die Leistung des Projektteams bei der Erreichung der angestrebten Projektziele? (Sehr niedrig 1-5 Sehr hoch) - Warum? - Was kann verbessert werden? |
| Challenges | - Have you faced any major setbacks/challenges in this project? What are they? - How did you feel at that time? (Emotions) - How did you (and the team members) react to these challenges? Why? (Reactions) - Do they have any significant impact on your work/ project performance? If yes, how? - Have these challenges been solved successfully? - If so, how? - If not, what do you plan to do? - What do you find most challenging about your work as a project leader in this (type of) project? Why? - What have you found to be important in helping you (or your team members) cope with the challenges? - Where do you see optimization potential? | - Sind Sie bei diesem Projekt jemals auf größere Rückschläge/ Herausforderungen gestoßen? Welche sind das? - Was empfinden Sie angesichts dieser Herausforderungen (Emotionen) - Wie haben Sie (und die Teammitglieder) auf diese Herausforderungen reagiert? (Reaktionen) - Wie wirken sich diese Herausforderungen auf Ihre Arbeit aus? Haben sie einen wesentlichen Einfluss auf den Erfolg dieses Projekts? Wenn ja, wieso? - Wurden diese Herausforderungen erfolgreich gelöst? - Wenn ja, wie? - Wenn nicht, was planen Sie zu tun? - Was empfinden Sie als größte Herausforderung bei Ihrer Arbeit als Projektleiter in diesem (Typ von) Projekt? Warum? - Was haben Sie als wichtig empfunden, um Ihnen (oder Ihren Teammitgliedern) zu helfen, die Herausforderungen zu bewältigen? - Wo sehen Sie Optimierungspotenzial? |

| Enablers | - What do you think is important for the effective implementation of the project? - How do you deal with this lack of…_? | - Was ist Ihrer Meinung nach wichtig für die effektive Durchführung des Projekts?   - Wie überwinden Sie diesen Mangel an _____? |
| --- | --- | --- |
| Open questions (5 minutes) | |  |
| Open questions | - From your point of view, what are the most important characteristics for project leaders to lead this type of project effectively? Why? - What message(s) or experience(s) would you share with those working on similar projects to yours/ working on projects similar to yours/? Why? | - Was sind nach Ihre Meinung die wichtigsten Eigenschaften für Projektleiter, um diese Art von Projekt effektiv zu leiten? Warum? - Welche Botschaft(en) oder Erfahrung(en) würden Sie gerne mit denjenigen teilen, die an ähnlichen Projekten wie dem Ihren arbeiten? Und warum? |
| Closing (5 minutes) | |  |
| Conclusion  Thank-you and closing of interview | - Any questions? - Would you like a copy of transcript? - Thank you for taking time off at this exceptional time. - If you have any further questions or wish to make any changes to your transcripts, please contact [Investigator X] at [E-mail]. - Stop recording. | - Haben Sie Fragen? - Kopie der Transkript? - Danke, dass Sie sich in dieser besonderen Zeit Zeit nehmen. - Wenn Sie weitere Fragen haben oder Änderungen an Ihren Transkripten vornehmen möchten, kontaktieren Sie bitte [X] unter [E-mail]. - Aufzeichnung enden. |

**Supplementary Material 3. Data Structures**

**
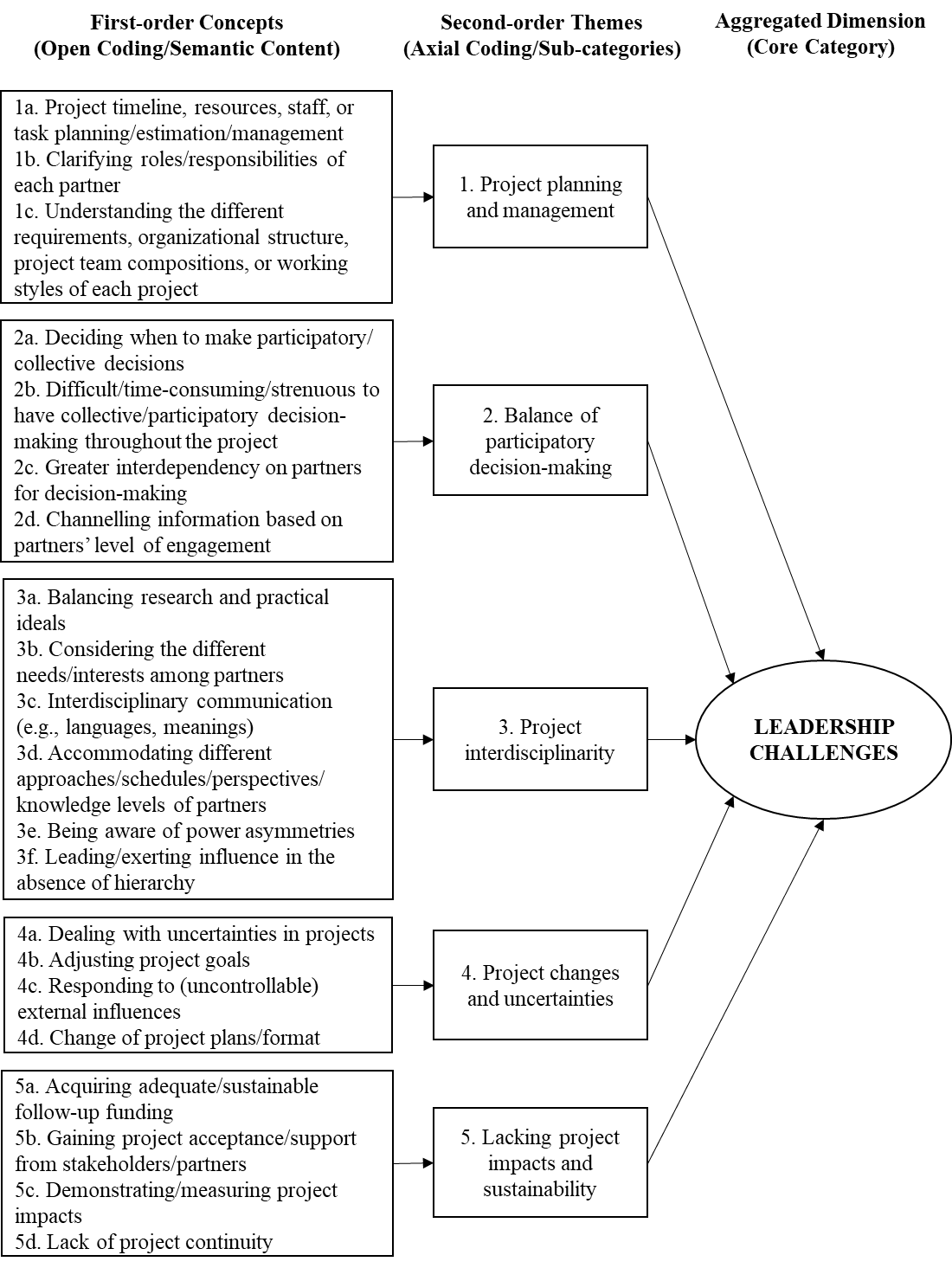
**


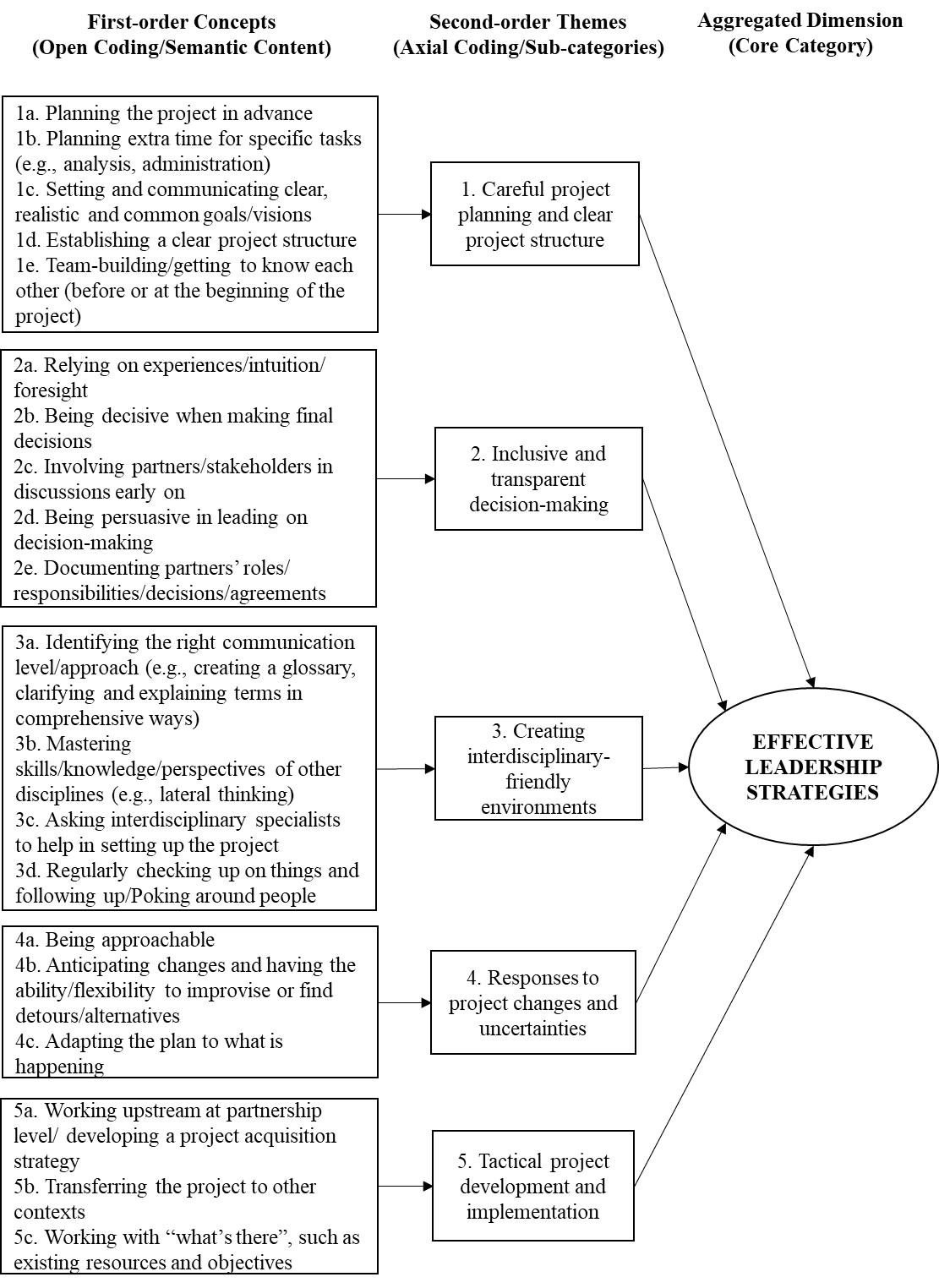


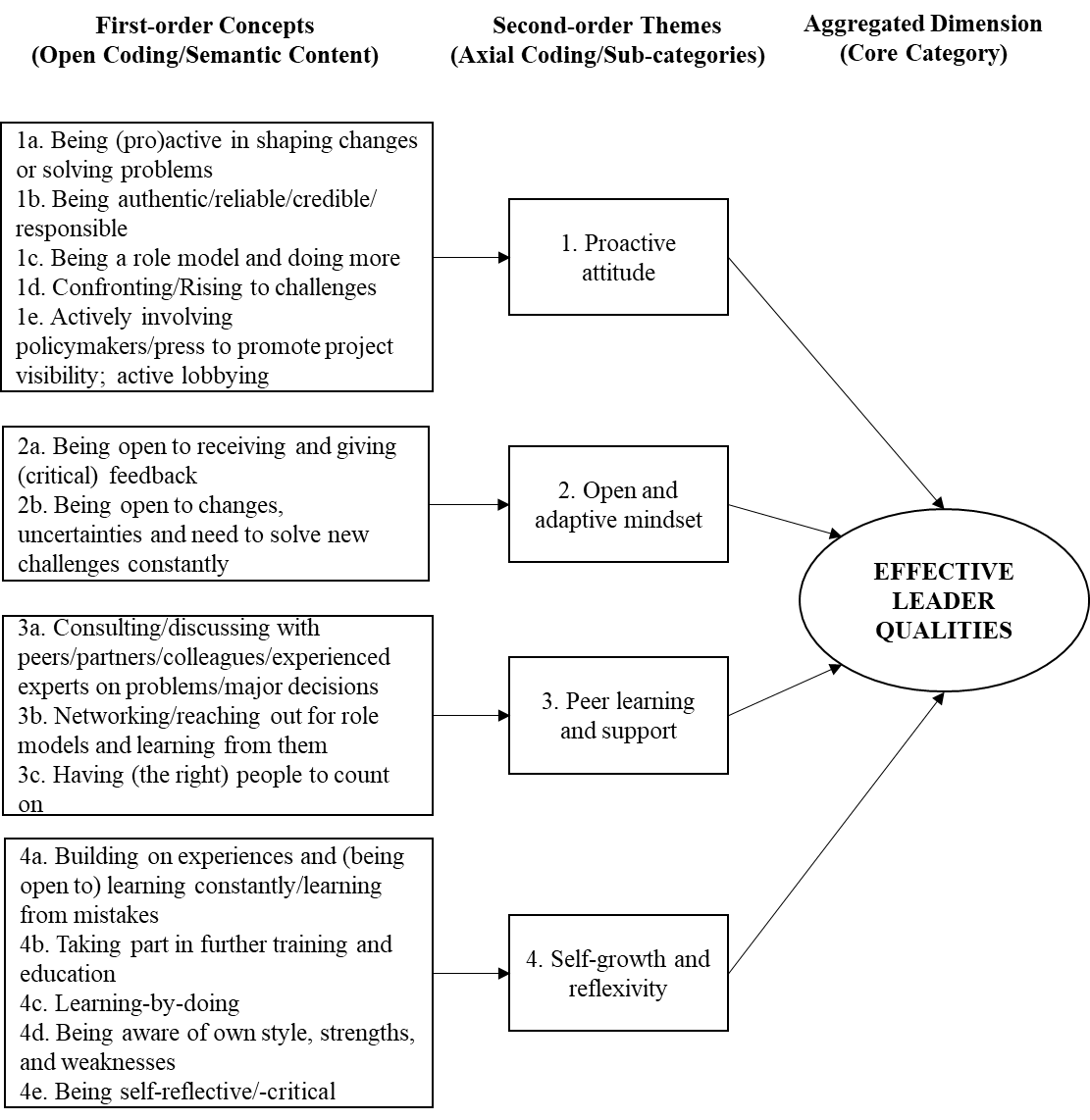


**Supplementary Material 4. Summary of Additional** **Quotes and Related Themes**

1. **Leadership challenges faced by CAHP project leaders**

| **Quotes** | **First-order Concepts**  **(Open Coding/Semantic Content)** | **Second-order Themes**  **(Axial Coding/Sub-categories)** |
| --- | --- | --- |
| “I think I was too relaxed and carefree. At the very beginning, I told the two employees that they should inform me regularly when they were working overtime and taking too many days off. And of course, I didn’t follow up on that and didn’t ask... when they started again, they surprised me with this wonderful news that they would now only work a few more days (laughs), and then I gave them the rest of the project off because they had so much overtime and holiday...To be honest, I was a bit annoyed that I had been too lax.” (Annie, 45, 5 Y.o.E) | **1a. Project timeline, resources, staff, or task planning/estimation/management** | **1. Project planning and management** |
| “…how to plan our human resources (...) was the big problem. We had a lot of administrative work, but we didn’t have so many people who could implement it. And there were deadlines behind it. This means that we had to decide at short notice to bring in more capacities, which had not been planned beforehand.” (Marie, 36, 2 Y.o.E) |  |  |
| “I said that it’s planning the events and managing the finances, the timelines and everything. Those are the big tests, no question.” (Janet, 31, 3 Y.o.E) |  |  |
| “…not only organizational, coordinating tasks in this whole complex, but also the financial administration is my big task.” (Lily, 34, 2 Y.o.E) |  |  |
| “More than it was the case before COVID, the project managers were really in demand, yes, to clarify things.” (Olivia, 29, 3 Y.o.E) | **1b. Clarifying roles/responsibilities of each partner** |  |
| “It always depends on the projects that you have and which members you have in the project.” (Elaine, 42, 4 Y.o.E) | **1c.** **Understanding the different requirements, organizational structure, project team compositions, or working styles of each project** |  |
| “This was also an unfamiliar approach for them, because other funding logics simply work differently than health insurance funding, both in science and in sports.” (Moses, 56, 2 Y.o.E) |  |  |
| “It depends on the project. ... Is that always successful? No. Would it not work in certain entities? Yes, absolutely.” (Natalie, 45, 10 Y.o.E) |  |  |
| “Well, a lot is going on at the moment, but when I see it for myself, I think it is the right mixture of participation and leadership.” (Iris, 35, 2 Y.o.E) | **2a. Deciding when to make participatory/collective decisions** | **2. Balance of participative decision-making** |
| "There are so many people involved, but you have to make sure you have everybody’s consent to decide what you want to decide, and you feel bad if you don’t (…) you always have to decide is that a thing that you can just decide by yourself? Or is it something you rather want to talk with your team about before you make any decision?" (Janet, 31, 3 Y.o.E) |  |  |
| “There are over twenty of us, we also have a board of directors, and they all have to make important decisions together, which is very, very difficult… So it was, it took a very long time back and forth to discuss how we should proceed and what measures we should or should not take. It was a very, very long time.” (Lily, 34, 2 Y.o.E) | **2b. Difficult/time-consuming/strenuous to have collective/participatory decision-making** |  |
| “Yes, the exciting thing is … there is no hierarchy in the sense that someone has the authority to give orders, but everything ONLY WORKS (emphasized) by consensus. On the one hand, this makes things a bit more strenuous; on the other hand, it also makes them relatively resilient.” (Moses, 56, 2 Y.o.E) |  |  |
| “Of course, we couldn’t influence that either. So, we relied on each other that the other practice partner or project partner would make the right decisions.” (Annie, 45, 5 Y.o.E) | **2c. Greater interdependency on partners for decision-making** |  |
| “Because once a consensus has been reached, no one leaves so easily.” (Moses, 56, 2 Y.o.E) |  |  |
| “To ensure that the information is filtered in such a way that it only reaches the relevant recipients; because the doctors told us relatively quickly that they do not want to be involved in the higher-level discussion processes. Rather they only want to have the results.” (Victor, 62, 6 Y.o.E) | **2d. Channeling information based on partners’ level of engagement** |  |
| “Yes, this is a cooperation project between a university and a practice partner, so you have the classic problem, the classic problem field of research practice that describes how problems or questions are often very often investigated in research, are not particularly relevant in practice; and on the other hand, practice does not work much with research findings, e.g., because they are difficult to understand or difficult to access. And this project is exactly at the interface between science and practice.” (Ron, 26, 2 Y.o.E) | **3a. Balancing research and practical ideals** | **3. Project interdisciplinarity** |
| “That is a relatively large consortium with very many practice partners. Many different organizational partners, and of course, the challenge has been to bring together practice and science. Above all, to bring together care and medicine, where there are still barriers on both sides.” (Victor, 62, 6 Y.o.E) |  |  |
| “I think that in scientific areas, like in chemistry where I come from, there is still a lot of lone wolves, and of course you have your teams with whom you work, but everyone stays a bit on their own. And that is not possible at all in such an innovation form project, for example.” (Karen, 30, 0 Y.o.E) |  |  |
| “And I’ve always said we’re doing research here. I don’t know what will come out. We have a question, and we investigate it. And we also check very carefully whether the question is relevant. But I can’t say beforehand whether it will work out afterwards. That’s just the way research is.” (Walter, 58, 6 Y.o.E) |  |  |
| “From the perspective of a scientist, … it has not been carried out with the scientific quality that one actually expects of oneself and where one also makes demands on oneself. So, that is actually, yes, that was the biggest hurdle for me. To come to terms with it.” (Annie, 45, 5 Y.o.E) |  |  |
| “In this particular project, the biggest challenge I have is to satisfy [the companies] because they’re paying me, but also to continue doing good work. And from my experience, if you work with the industry, that also works much better, but that’s my personal experience. So, when, but that was in the States, when we worked with, let’s say [company name], they just gave you money. Of course, I know what they want. But they let you do your thing. And they don’t say, “Well, how about we add this and this?” And I’m thinking, “No, we’re not adding that.” Yeah, so, but I think this is very particular to that project, and so that’s one challenge.” (Natalie, 45, 10 Y.o.E) |  |  |
| “The characters are very different anyway. You have that everywhere in life. But I also believe that different types of people find different things interesting and important, and therefore position themselves differently in their work and their job, and as that’s where things collide, of course.” (Bonnie, 35, 2 Y.o.E) | **3b. Considering the different needs/interests among partners** |  |
| “And … we only have projects with other actors, and they all have other tasks and often other interests. This is extremely widespread in the health system. That’s why the interests are vital.” (Walter, 58, 6 Y.o.E) |  |  |
| “It’s a constant struggle… And when something is different, you’re lucky if people like it. But then there are at least many people who find it bad, of course, because it was different before… This is extremely widespread in the health system.” (Walter, 58, 6 Y.o.E) |  |  |
| “Many things are not possible because the interests are too different.” (Moses, 56, 2 Y.o.E) |  |  |
| “Probably, if you have a … collaborative leading style, then you usually don’t get one opinion from all the team members. They have different opinions, which is okay. Still, then there has to be a decision and to decide while knowing that some people do not agree with you, can be hard, I think because you want everybody to be happy and you do things that you think is best for the results. And that might be, I don’t know if that’s the hardest thing, but that can be something.” (Janet, 31, 3 Y.o.E) |  |  |
| “There are still scientists with me who can’t explain to computer scientists what they are actually supposed to build… So that’s interdisciplinarity, and it doesn’t usually happen. That’s my firm conviction because most of them can’t even get into the heads of the others.” (Walter, 58, 6 Y.o.E) | **3c. Interdisciplinary communication (e.g., languages, meanings)** |  |
| “I also noticed that I had reached my limits in terms of communication. So, I had to learn foreign languages, so to speak, and had to ask a lot of questions: ‘Explain this to me.’” (Moses, 56, 2 Y.o.E) |  |  |
| “So, I think it was a much greater challenge for them because you have to say that depending on the project... they have very different approaches. Some of them simply didn’t have the financial means or technical requirements. Therefore, it was really a big challenge.” (Kelly, 50, 4 Y.o.E) | **3d. Accommodating different approaches/schedules/perspectives/ knowledge levels among partners** |  |
| “If you have funds available in your budget to finance this, you can reallocate funds. Or how does that work? This is a topic that has to be discussed repeatedly, wherever it is necessary to consider that the financial structure of the original organization is different.” (Sophie, 54, 9 Y.o.E) |  |  |
| “I don’t know how much they had to do with it… I need the keys again to combine the right files relationally. They didn’t do that. So afterwards we all grabbed our heads, they did too.” (Walter, 58, 6 Y.o.E) |  |  |
| “What was more challenging? Yeah, it’s a different approach from the different professionals… I think the others have a more specific view on things, and I think this was a bit different from talking to each other with this different view.” (David, 42, 4 Y.o.E) |  |  |
| “However, it was forgotten that strong power asymmetries always mark these intercultural encounters and that one is usually the host, and the other is thus the one who is tolerated for once and allowed to be there.” (Carla, 49, 5 Y.o.E) | **3e. Being aware of power asymmetries** |  |
| “And these power relations are the subject of discussion when it comes to the concept of eye level. We discussed these very strongly with the students because they were not at all aware that there were power differences between them and the residents.” (Carla, 49, 5 Y.o.E) |  |  |
| “Everyone works according to his/her abilities. Moreover, the person who has the overall project in hands is open to everything that comes from all sides, irrespective of the fact that people now say that I am the project manager.” (Helen, 50, 10 Y.o.E) | **3f. Leading/exerting influence in the absence of hierarchy** |  |
| “So, we are now submitting the applications, which is a good time to get the money; but we don’t know if we can start the process now as at the moment there are no applications (...), which is difficult for our department when people are no longer allowed to meet.” (Jasmine, 35, 2 Y.o.E) | **4a. Dealing with uncertainties in projects** | **4. Project changes and uncertainties** |
| “So even then, there was the fear of: ‘how long is all this going to take? Can I even manage that? Do I have to change projects?’. That was also a huge uncertainty factor for the clinical staff and me. It was simply a complete overload and overwork because we suddenly had a lot more to do than before.” (Lily, 34, 2 Y.o.E) |  |  |
| “Yes, we had a relatively difficult start because the project didn’t start (...), we wanted to postpone it three months because it was in the middle of the summer holidays and a team should somehow be founded... it was actually not possible to communicate with the administration, where everyone is on vacation...And then we started later. The start was a bit difficult.” (Nelson, 46, 14 Y.o.E) |  |  |
| “We have put three years of work into it and have really been very diligent. But now we basically don’t know whether it is really as effective as we originally imagined.” (Annie, 45, 5 Y.o.E) |  |  |
| “And that’s what we are trying to figure out: how to do it or if we can do it sometime later because no one knows what to expect in a few months.” (Janet, 31, 3 Y.o.E) |  |  |
| “We have, of course, adjusted some goals, maybe even reduced them and simply said, ‘Okay, we will not have measured so many people in September. That will not be realistic. We must now set realistic new targets to take the pressure off a bit.” (Lily, 34, 2 Y.o.E) | **4b. Adjusting project goals** |  |
| “Yes, we were forced to do some tasks we had planned to do, which were also included in the project plan. We did indeed get rid of some tasks. We put them down, I would say, we could not fulfil them anymore... In some subprojects, which were very strongly based on empirical research methods, we had to make cutbacks.” (Max, 68, 20 Y.o.E) |  |  |
| “Some of the basic work has been moved online, but some things have actually simply been moved and replaced.” (Nelson, 46, 14 Y.o.E) |  |  |
| “If there are external influences, where you come to the conclusion that the vision has to be changed, or maybe it has to be discarded, or the project ends for this; that is, of course, manslaughter. Nothing can be done about that. You just have to do it in such a way that you have to say okay, you have to look for alternatives or go in a completely different direction and redefine it completely.” (Elaine, 42, 4 Y.o.E) | **4c. Responding to (uncontrollable) external influences** |  |
| “In the hospital world, for example, political decisions are often made that force you to re-prioritize, where suddenly a deadline comes along, which was not so clear before, or ... one has to react and say: ‘Okay, we have to let this project rest for now and something give higher priority to other things’.” (Olivia, 29, 3 Y.o.E) |  |  |
| “This year, we are a little behind with the management of clients or with the admission of clients due to the lockdown; and of course, also because here we could not send so many clients there.” (Sophie, 54, 9 Y.o.E) |  |  |
| “So, we still try to develop these noninvasive imaging techniques, and we try to keep this direction. But unfortunately, we are also dependent on projects funded by third parties, and we may be looking at what is coming. And so sometimes we have to adapt this vision to practical problems, such as financial problems.” (Daisy, 46, 4 Y.o.E) | **4d. Change of project plans/format** |  |
| “Some tasks cannot be done. For example, in the project... it was planned that we make admissions on patients now. This is of course not possible anymore, but the project is not finished either.” (Daisy, 46, 4 Y.o.E) |  |  |
| “And that means that I should have started in spring, that was the time when everything was still completely unclear when I was, of course, a bit behind in my original plan and we decided ‘Okay, we won’t postpone this symposium one month later, we’ll just cancel it completely’… I simply said it doesn’t make any sense at the moment and it will take place in March of next year, but then it was postponed again.” (Kelly, 50, 4 Y.o.E) |  |  |
| “And I think only now has everyone understood that it won’t go away, COVID will not go away, for the time being, it wasn’t just a three-week lockdown. Now we have to change everything slowly and live with the fact that our goals will not be reached as planned.” (Lily, 34, 2 Y.o.E) |  |  |
| “Yes, we have an important final meeting, we had to cancel it completely. Maybe we could have organized it as an online conference as well. But … I think it is difficult to organize an intensive conference with 40 people. That was difficult. We did not dare to do that anymore.” (Max, 68, 20 Y.o.E) |  |  |
| “Some of the basic work has been moved online, but some things have simply been moved and replaced.” (Nelson, 46, 14 Y.o.E) |  |  |
| “We applied for a third-party funding and submitted it to various project promoters...it was quite a challenge to get money for this project.” (Anna, 53, 12 Y.o.E) | **5a. Acquiring adequate/sustainable follow-up funding** | **5. Lacking project impacts and sustainability** |
| “There is a list of all these research associations and, um, they will all try to make new applications, most of them. I think two want to stop, and there is only a limited amount of money to be distributed. Therefore, not everyone who would like to can continue.” (Bonnie, 35, 2 Y.o.E) |  |  |
| “We have already seen a few developments where project partners then said, ‘I didn’t apply for funding for this. Now I have to check in my organization to free up funds for this.’ Yes, these are financial hurdles we are already facing.” (Sophie, 54, 9 Y.o.E) |  |  |
| “The positive public perception of this project is also important.” (Queenie, 64, 22 Y.o.E) | **5b. Gaining project acceptance/support from stakeholders/partners** |  |
| “Because of the first lockdown, of course, I would say target group’s acceptance...are not as far as we wanted to be today.” (Sophie, 54, 9 Y.o.E) |  |  |
| “But other actors in the health care system also have to accept that there is something new there that is not in competition with them, rather that we have initiated the project to close a gap and form a loophole, and you have to advocate it a lot and communicate a lot to achieve this acceptance.” (Tina, 55, 5 Y.o.E) |  |  |
| “This is very, very difficult. So, uh, we have done a quantitative study and generated a lot of soft, subjective criteria. So, what we can say in any case is that the degree of dementia has not worsened under this intervention but has improved slightly...we could not make any significant statements. However, we could clearly see many, many emotional improvements, also in the situation.” (Anna, 53, 12 Y.o.E) | **5c. Demonstrating/measuring project impacts** |  |
| “That there is another assumption that, of course, there would be even more severe cases. So, we could not make any significant statements. However, we could see many, many emotional improvements, also in the situation; so, we can tell stories about it, for example, that, hmm, one session was about making a hay bed and in the evening a resident told his caring nurse, oh he would like to sleep in the hay bed today. These were things that, uh, where he would otherwise never be able to remember much about the day’s activities” (Anna, 53, 12 Y.o.E) |  |  |
| “Well, we measure it by the resonance. For example, the speaker, who has a lot of experience in open senior citizens’ work, came to us and said she had never experienced anything like this before. So many things disturbed her in other cafés and had never happened to any of us. And such resonances naturally make us understand how special the whole thing is. Otherwise, we would not even think that it is so successful. Simply the many positive feedbacks and that we have the feeling all the time that it is going so well.” (Iris, 35, 2 Y.o.E) |  |  |
| “It is still exciting to see whether we will succeed because the goal we have set ourselves is to get five hundred people in [city name] in five years to actively work on their [health condition], to get involved in the network, and to achieve a certain percentage of 20%... But in the end, the project is really about the people in work, about having resources, i.e., having fought the [health condition], about having been socially integrated, about having social and professional participation, and about being happy to make this possible.” (Sophie, 54, 9 Y.o.E) |  |  |
| “Yes, of course, that’s a difficult topic because I think it takes a lot more time. That’s not something you can just come up with in two years because, of course, early warning systems are always, you need a specific reason, no, like now, for example, the spread of the virus…Um, I would say that it still needs a bit more time before you actually have tangible results.” (Bonnie, 35, 2 Y.o.E) | **5d. Lack of project continuity** |  |
| “Well, the vision is that it can be continued sustainably, but it is difficult, and we have encountered obstacles that we cannot influence…There is nothing more I can do; I can no longer pursue my vision. And then you just have to look for other projects.” (Elaine, 42, 4 Y.o.E) |  |  |
| “That is the difficulty, yes, so actually they should have a follow-up project in 5 years, where the statistical analysis of health care is repeated. And then to see whether the whole system is in a better position.” (Paul, 55, 14 Y.o.E) |  |  |

1. **Effective leadership strategies**

| **Quotes** | **First-order Concepts**  **(Open Coding/Semantic Content)** | **Second-order Themes**  **(Axial Coding/Sub-category)** |
| --- | --- | --- |
| “... from project planning [laughs], where you simply learn a lot now. What you should have planned differently and what you should have applied for differently in the project implementation.” (Claire, 40, 6 Y.o.E) | **1a. Planning the project in advance** | **1. Careful project planning and clear structure** |
| “I would recommend that the project management team define the project team and its roles right from the start so that you don’t end up in the predicament of not having enough staff or the right people in the project. You have to think about who you need beforehand.” (Marie, 36, 2 Y.o.E) |  |  |
| “Take a lot of time in preparing the project ... especially for the project application.” (Moses, 56, 2 Y.o.E) |  |  |
| “I would plan a lot more time because it makes a lot of sense to accompany the research. Even if you research in your community, it’s always good to have an exchange room for reflection and questions.” (Jasmine, 35, 2 Y.o.E) | **1b. Planning extra time for specific tasks (e.g., analysis, administration)** |  |
| “Probably plan the research better, apply for the budget for the interviews and translations, plan more time.” (Jasmine, 35, 2 Y.o.E) |  |  |
| “I think communicating a vision and formulating it together is important at the beginning of the project. It is important not to discuss the vision and not to propagate it all the time, but it is essential to define which tasks each partner has to realize the vision.” (Elaine, 42, 4 Y.o.E) | **1c. Setting and communicating clear, realistic, and common goals/visions** |  |
| “In the past, I completely underestimated how important the topic of mission and vision is. We have an evident vision: we want to provide patients in intensive care units with a personalized intervention… This is something that we communicate very vehemently and clearly to everyone. Everyone has to understand it... because the shared vision keeps the team together.” (Gary, 40, 5 Y.o.E) |  |  |
| “I think it is important to exchange a lot of information with the different people to find out their motives and interests, to know them well, and to find common points of contact or overlap. These are then communicated to the respective team members in a way that if I want to push through a topic or a research question, for instance, I can explain on both sides why this research question is important and why we are pursuing it now.” (Ron, 26, 2 Y.o.E) |  |  |
| “We all had the declared aim of implementing this project, so everyone who took part wanted to do something for this target group… that was already clear to all of us.” (Sophie, 54, 9 Y.o.E) |  |  |
| “We started with a smaller vision...we have become bigger and broader…the clear focus is more on competitiveness and job creation in Germany. Of course, we cannot start with an extremely free vision and hope that in 10 years, we will have something. That is not possible, logically. We must certainly also carry the vision and have a clear focus on product development.” (Morton, 45, 4 Y.o.E) |  |  |
| “How we set the common goals... it’s a lot of talking also to each other and new members who develop different language levels, even have changed the goals a bit so that everybody can say: ‘Okay, there’s a bit of me in these goals’.” (David, 42, 3 Y.o.E) |  |  |
| “On the one hand, giving people a lot of freedom. On the other hand, standing by your words and telling people: you have to stand by your actions...That means setting the framework, offering help and giving freedom.” (Gary, 40, 5 Y.o.E) | **1d. Establishing project structure** |  |
| “The project managers were really in demand… to clarify things and put things into a structure.” (Olivia, 29, 3 Y.o.E) |  |  |
| “What responsibility does everyone have, and what responsibility does the project manager have? In other words, a fixed or predefined structure from the outset that you can rely on. I think that’s the most important thing. And then, a lot can be decided individually within that.” (Marie, 36, 2 Y.o.E) |  |  |
| “Meetings should be paired with some kind of team-building measures, like, how do we get together.” (Anna, 53, 12 Y.o.E) | **1e. Team building/getting to know each other (at the beginning of the project)** |  |
| “Before you even start working on the project, have a long preparatory phase where you talk a lot or have a cup of coffee or tea with each other. In other words, a phase of getting to know each other.” (Moses, 56, 2 Y.o.E) |  |  |
| “Of course, in such a big project it also costs a lot of time and costs, one has to say, if the personnel of five partners always travel throughout Germany. Nevertheless, I think the personal connection is critical. At least at the beginning of such a meeting, you plan it somehow.” (Claire, 40, 6 Y.o.E) |  |  |
| “We first had to adjust to each other, i.e., in a large project consortium, when a total of, let’s say, 30 to 40 people are involved in some way… These people first have to get to know each other and get used to each other. And that was not easy for many at the beginning... So, on the personality level, I think we had to learn a lot first.” (Max, 68, 20 Y.o.E) |  |  |
| “I would try to get all the people involved together faster so that everyone could talk about their different goals, views and interests related to the project, such that they could be at the same level. I would also maintain this format at regular intervals so that everyone knows what is going on in the other institutions and what interests they have to avoid coordination problems that would otherwise arise.” (Ron, 26, 2 Y.o.E) |  |  |
| “I have more foresight, I think, and know what is truly important.” (Lily, 34, 2 Y.o.E) | **2a. Relying on experiences/intuition/foresight** | **2. Inclusive and transparent decision-making** |
| “You naturally gain personal experience.”  (Tina, 55, 5 Y.o.E) |  |  |
| “The decisive thing is to develop a feeling for it simply - how should I communicate, so that my counterpart can take the messages up accordingly? What is he/she used to?” (Victor, 62, 6 Y.o.E) |  |  |
| “When you are that old, you can build on your experience, and you are constantly learning. And I think that was an important asset for me.” (Barry, 64, 9 Y.o.E) |  |  |
| “Um, intuition (laugh)… there are some, well, not rules, but things you just have to do in a certain kind of way. But other than that, I just do it as in the way I think it’s right.” (Janet, 31, 3 Y.o.E) |  |  |
| “When you have so many partners, you naturally want to make decisions together, and decision-making committees have also voted on things… however, it is still important for a project manager to be able to make decisions. If it comes to the fact that there are problems… you have to hit the table and decide.” (Elaine, 42, 4 Y.o.E) | **2b. Being decisive when making final decisions** |  |
| “You have to have one person who makes the final decision, and that’s always me in the case of critical decisions, in the case of unclear decisions. Because somebody has to have the final say in the end.” (Gary, 40, 5 Y.o.E) |  |  |
| “Within the project, I make the final decision…but of course, the decision is also based on discussions with the project partners, cooperation partners and conventional partners. I try to work as transparently as possible.” (Karen, 30, 0 Y.o.E) |  |  |
| “What I have learned concretely is that we have waited too long to address a crisis. We have learned that you have to deal with crises as soon as they occur or if there are little things like that, the whole thing needs to be discussed.” (Anna, 53, 12 Y.o.E) | **2c. Involving partners in discussions/solving crises early on** |  |
| “I would do that differently right at the beginning of the project and describe to the other persons to get an idea of what is my ability and what is yours.” (Anna, 53, 12 Y.o.E) |  |  |
| “At the very beginning, we had planned a first project meeting with all partners, but the disadvantage of these projects is that there is a starting point [of the project].” (Claire, 40, 6 Y.o.E) |  |  |
| “When you have so many partners, you naturally want to make decisions together… however, it is still important for a project manager to be able to make decisions. If it comes to the fact that there are problems… you have to hit the table and decide. But in principle, it is of course important to involve the partners on an equal footing, to explain things to them, to make things clear, to be transparent and then come to a joint decision, which hopefully is in everyone’s interest and of course in the interest of the project management too.” (Elaine, 42, 4 Y.o.E) |  |  |
| “The others were not anchored because we did not take them all along from the beginning. So, in the application process, I would involve the local actors even more.” (Carla, 49, 5 Y.o.E) |  |  |
| “Anything that could be questionable in any way is simply thrown into the discussion without delay...In the meantime, we have developed a culture of checking with the others: ‘Is what I’m about to do okay? Do you see it the same way?’” (Moses, 56, 2 Y.o.E) |  |  |
| “I think that my many years of experience have taught me how to use statistics - I have to sell.” (Paul, 55, 14 Y.o.E) | **2d. Being persuasive in leading on decision-making** |  |
| “This is persuasion work I have to do, thanks to the fact that I am dealing with the different people and interests in the team.” (Ron, 26, 2 Y.o.E) |  |  |
| “There were regular minutes written at the meetings and partly also at the conference calls. These transcripts were made available to everyone. Um, so that when people looked at the minutes, they could have the same level of knowledge at all times, right? Not everyone does that, of course. One is more actively involved than the other. However, theoretically, anyone could have known the same thing as anyone else at any time. And that has proven to be very helpful.” (Bonnie, 35, 2 Y.o.E) | **2e. Documenting partners’ roles/responsibilities/decisions/agreements** |  |
| “Another success factor: documentation. I just said I always keep everything. That’s super important because you can be quite sure that it was completely different after a year. Suppose you can no longer document how it was. The people who say that don’t mean it badly. They just have it figured out in their heads. That means cognitive dissonance reduction, yes, a very, very important human behavior. But it’s not true. No, in fact, it was agreed, ‘see here mail from so-and-so, and letter from so-and-so’. Sometimes I drive my people here crazy when I always say ‘document’. We keep a project diary in every project, where we write something down every day… That is a crucial success factor. By the way, it’s also a quality factor. Otherwise, you do something else after half a year.” (Walter, 58, 6 Y.o.E) |  |  |
| “That is only possible by constantly exchanging ideas, communicating. If possible, record what has been communicated through minutes. We followed a different solution. We always recorded it afterwards and then sent it to everyone by E-mails so that even if someone was not there, they were also informed. And then, you always had something like a bullet point that you could refer to again. There’s no other way, but I don’t think that has anything to do with Corona...The more people are involved, the more important it is that everyone is always on the same level and knows what it’s all about.” (Annie, 45, 5 Y.o.E) |  |  |
| “Even though I described the administration effort as a challenge, I would say that documentation contributes to more transparency for yourself and all those involved in the project. This means, for a decision, also a joint decision (laughs), we take minutes and notes for everything, for every conversation… It doesn’t have to be that elaborate, but somehow what was discussed must be documented.” (Marie, 36, 2 Y.o.E) |  |  |
| “And at the end, which is a bit banaler, but good documentation of what has been agreed. That is also very important.” (Moses, 56, 2 Y.o.E) |  |  |
| “To create a kind of glossary...a kind of dictionary, where the most important six or seven technical terms they would use were described in simple words. What’s that about? And what do I want to achieve with it? And what does it mean?” (Bonnie, 35, 2 Y.o.E) | **3a. Identifying the right communication level/approach (e.g., creating a glossary, clarifying, and explaining terms in comprehensive ways)** | **3. Interdisciplinary-friendly environment** |
| “I think it is always important to find a level of language and a level of communication that enables us to present our issues in a comprehensible way, that we are understood with our concerns.” (Barry, 64, 9 Y.o.E) |  |  |
| “We succeeded in making it understandable, comprehensible and plausible so that the project…was also assessed as feasible and fundable…You have to talk to the people [partners]. You have to try to find out what, say, what is wanted and required? Well, you also have to fulfil certain requirements. And then, you have to see if you can somehow manage it together.” (Barry, 64, 9 Y.o.E) |  |  |
| “I believe that all the important solutions lie at the boundaries of the disciplines. That is also where we have achieved our greatest successes, and other great successes are almost always at the borders of subjects, not at the centre. At least in health care, that is the case.” (Walter, 58, 6 Y.o.E) |  |  |
| “The technical language can be learned - that’s not the problem. However, many terms appear in every field, but the meanings are specific in each field. And that can cause a lot of trouble if you don’t clarify the differences.” (Moses, 56, 2 Y.o.E) |  |  |
| “It is vital that you include individuality in your management style and shows real understanding… and also accept the different ways of working. And I think that this can also be transferred well to national team leadership, i.e., that you look at each individual as an individual, and you know what the other person’s background is, what he or she has experienced, how their training went, or any possibilities influencing an individual’s working methods.” (Elaine, 42, 4 Y.o.E) | **3b. Mastering skills/knowledge/perspectives from other disciplines (e.g., lateral thinking)** |  |
| “We first had to adjust to each other… these people first have to get to know and get used to each other.” (Max, 68, 20 Y.o.E) |  |  |
| “I think that knowledge was fundamental here. At least to have a bit of a medical background, so to speak, and to be able to assess what is happening and also to be able to explain it to the team members.” (Olivia, 29, 3 Y.o.E) |  |  |
| “I have to understand at least enough to be able to explain what [the partner] is supposed to do. And [the partner] should understand so much that he knows what I need in the end... And at the interfaces, you really get into...That’s where it gets adventurous.” (Walter, 58, 6 Y.o.E) |  |  |
| “Ultimately, you should also master the skills and abilities that are required of others. I think that’s important.” (Annie, 45, 5 Y.o.E) |  |  |
| “Right at the beginning... we decided that we would get support and hired two people from a university who know about interdisciplinary work. They have always come to our meetings and listened, for example, how do we communicate? How is that received by everyone?... which worked quite well.” (Bonnie, 35, 2 Y.o.E) | **3c. Asking interdisciplinary specialists to help in setting up the project** |  |
| “I regularly check up on things and do the follow-up. ‘Man, what’s up? You got all the records you need already? Can you expand that already? You don’t have the data sets. Why is that?’… That means I have to poke around. Well, I can’t decide how quickly he or she’s going to deal with anything. But I have to ask in time to find out why it’s not going well.” (Helen, 50, 10 Y.o.E) | **3d. Regularly checking up on things and following up/poking around people** |  |
| “You have to be approachable. You have to be accessible, especially when these are projects that span several working groups, whether the project is running in several working groups or even several locations. You must regularly look into your e-mails and answer things, even if you have the feeling that I don’t have time for that right now.” (Helen, 50, 10 Y.o.E) | **4a. Being approachable** | **4. Responses to project changes and uncertainties** |
| “I’m always there for everyone.” (Walter, 58, 6 Y.o.E) |  |  |
| “Yeah, maybe another part is that my team, my two colleagues, and I are quite close to the other teams, we talk a lot, we know what they are doing right now. We know their plans, their visions and what they’re going to do during the upcoming weeks or months.” (Janet, 31, 3 Y.o.E) |  |  |
| “What you learn over time, also independently of the pandemic, is the skills to improvise, that something can’t be controlled directly - for that, you have to find detours.” (Max, 68, 20 Y.o.E) | **4b. Anticipating changes and having the ability/flexibility to improvise or find detours/alternatives** |  |
| “Keep calm, analyze, look for alternative approaches, stay transparent, inform all project partners about these changes.” (Nelson, 46, 14 Y.o.E) |  |  |
| “This shift of work...the medium of communication has shifted much more. There are simply different communication channels and information flows. That is what has massively changed and also this admission. Okay, let’s take care of something else, react flexibly now.” (Nelson, 46, 14 Y.o.E) |  |  |
| “This also goes hand in hand with patience and, as they say, resilience. In other words, having a bit of sensitivity for the fact that some things may not go as you would like.” (Marie, 36, 2 Y.o.E) |  |  |
| “We are a bit behind schedule, as it always is. But not dramatically, because the evaluation of the research took longer than planned. But you make a plan to adapt it to what is happening.” (Jasmine, 35, 2 Y.o.E) | **4c. Adapting plans to what is happening** |  |
| “I think it would be easy for the statistical analyses if you didn’t just have a sub-project, but that this was a project, which would be totally upstream.” (Paul, 55, 14 Y.o.E) | **5a. Working upstream at partnership level/developing a project acquisition strategy** | **5. Tactical project development and implementation** |
| “It would be good if the idea could be disseminated or transferred to other contexts because it is simply more sustainable.” (Jasmine, 35, 2 Y.o.E) | **5b. Transferring the project to other contexts** |  |
| “This is precisely the participatory aspect - by working with people and, of course, with the resources available.” (Jasmine, 35, 2 Y.o.E) | **5c. Working with “what’s there”, such as existing resources and objectives** |  |

1. **Effective leader qualities**

| **Quotes** | **First-order Concepts**  **(Open Coding/Semantic Content)** | **Second-order Themes**  **(Axial Coding/Sub-category)** |
| --- | --- | --- |
| “If you keep getting angry about it and ask yourself why others aren’t doing it, nothing will come. And I think in the role of project management, you are very well advised to address the problems directly from the outset. And look for a solution together or try to weigh up what can be given for the most part.” (Marie, 36, 2 Y.o.E) | **1a. Being (pro)active in shaping changes or solving problems** | **1. Proactive attitude** |
| “I was very much in the role which had to demand information proactively because otherwise, I wouldn’t have got it right.” (Moses, 56, 2 Y.o.E) |  |  |
| “A sense of responsibility was already essential here. But as a rule, I would say that it is bad if a project manager is not responsible because, in the end, all the threads always come together.” (Olivia, 29, 3 Y.o.E) | **1b. Being authentic/reliable/credible/responsible** |  |
| “If you see yourself more as a team player, then you can also take up other aspects from other directions... it causes a great acceptance of the project. I can only lead successfully if my partners accept me as a leader and accept the whole project and the approach. And when I communicate and cooperate, it also means that I sometimes accept counterproposals, take them seriously and implement them under certain circumstances. And that is an instrument to make partners feel valued and feel taken along.” (Tina, 55, 5 Y.o.E) |  |  |
| “If you have a bad image, you are lost. In my opinion, credibility is the be-all and end-all for projects of a certain size.” (Walter, 58, 6 Y.o.E) |  |  |
| “Authenticity, so that people buy that I want this project personally, that is, I think, quite decisive.” (Moses, 56, 2 Y.o.E) |  |  |
| “This is the most important thing, if the leader is not a role model, who else is?” (Gary, 40, 5 Y.o.E) | **1c. Being a role model and doing more** |  |
| “Instead, leadership ability is characterized precisely by the fact that you are confronted with new challenges and new issues, almost constantly and have to solve them …” (Tina, 55, 5 Y.o.E) | **1d. Confronting/rising to challenges** |  |
| “We also had a very committed journalist from the sponsor with us who wanted to report on the project. Normally, we share our vision with the community. This is done by the association from [city name] because they originally initiated the project so that we also use a platform beyond that.” (Helen, 50, 10 Y.o.E) | **1e. Actively involving policymakers/press to promote project visibility; active lobbying** |  |
| “All the press work that we do. So, I think that many citizens in [city name] have heard that before. And many people are involved. We currently have a donor base of about 2,000 people…And for companies, so it is all together. That means, of course, that also radiates back to the entire company. If we have positive personal relations, then there it is. It radiates positively on the [hospital name] and certainly the [company name] in general. So that draws circles, so to speak.” (Queenie, 64, 22 Y.o.E) |  |  |
| “we always advocate… And many journalists, including radio and television, come and are interested in this kind of project.” (Walter, 58, 6 Y.o.E) |  |  |
| “But we are out there and say, ‘Please join our project,’ because we need another 500 patients for a large intervention study, and this issue of interest is already solid. So, we work very intensively with the state government and the federal government in various groups and working groups. We have already influenced laws several times. In some cases, it even says that we have done so, also at the federal level.” (Walter, 58, 6 Y.o.E) |  |  |
| “You have to be able to share ideas yourself. You have to be able to take it. So, then you have to be able to take criticism from others.” (Elaine, 42, 4 Y.o.E) | **2a. Being open to receiving and giving (critical) feedback** | **2. Open and adaptive mindset** |
| “What you learn over time, also independently of the pandemic, is the skills to improvise, that something can’t be controlled directly - for that, you have to find detours.” (Max, 68, 20 Y.o.E) | **2b. Being open to changes, uncertainties and the need to solve new challenges** |  |
| “You don’t have tasks that have to be carried out by always following the same procedures. Instead, leadership ability is characterized precisely by the fact that you are confronted with new challenges and new issues, almost constantly and have to solve them.” (Tina, 55, 5 Y.o.E) |  |  |
| “When they are no longer full members, they remain with us as associate members. In other words, they are still interested in taking part and getting the information on how things are going.” (Bonnie, 35, 2 Y.o.E) | **3a. Consulting/discussing with peers/partners/colleagues/experienced experts on problems or complicated/major decisions** | **3. Peer learning and support** |
| “We also have the [health department] in the background, which also advises us in such matters, and which also gives us a few suggestions, where we orientate ourselves on what we then adapt to our project.” (Jasmine, 35, 2 Y.o.E) |  |  |
| “I would like to know if the other projects have that [challenge] as well and if they feel the same way.” (Morton, 45, 4 Y.o.E) |  |  |
| “Especially when it’s more complicated things, where decisions have to be made about direction or perspective, strategic questions. We often have: ‘Are we going to do this at all or how are we going to do it?’ or ‘How will the next project go?’. You always have to think about the next project and the one after that with these third-party funded research projects. And that’s the kind of thing you have to discuss with the colleagues involved. That’s how we do it.” (Walter, 58, 6 Y.o.E) |  |  |
| “But before that, there were also many years where I was always the contact person alone. There was no intermediate layer. And then there were the informal ones because there were certainly always more experienced people whom we could ask questions.” (Walter, 58, 6 Y.o.E) |  |  |
| “I’m also really, really lucky that I have a relatively large number of quite clever people working for me. And I always consult with them.” (Walter, 58, 6 Y.o.E) |  |  |
| “We were part of a network, so within the network, there was always an exchange and the opportunity to talk about problems and challenges, especially with Corona, which was of course very helpful.” (Annie, 45, 5 Y.o.E) |  |  |
| “Well, actually, I wasn’t the only project leader. There was also [person X]’s boss, who was formerly the project leader... And that was sometimes quite helpful that I had regular meetings with her where we talked about the project. That was always another perspective from outside, from her. And since we had a long tradition of collaboration and cooperation and know exactly what makes each other tick, that was also quite well to talk about: "Where are we right now? Where are the problems, where are the difficulties? How can we tackle them?” (Annie, 45, 5 Y.o.E) |  |  |
| “We discussed it together and consulted with [partner name], which then also discussed the hygiene concept with the [city name]. Yes, we had an unbelievable number of consultation situations and discussed it repeatedly. In the end, I think we thought more about it than the participants themselves.” (Annie, 45, 5 Y.o.E) |  |  |
| “Well, I definitely have to say that it’s really, really good to have a team and to be not one person who has to decide everything. So, my two colleagues and I can talk about everything. I mean, yes, everybody has her tasks. I have to say ‘her’ because we are only girls, but we can also talk about it. And I can say, well, I would like to do this, but I’m not sure. What do you think about it? And that really helps in critical situations?” (Janet, 31, 3 Y.o.E) |  |  |
| “You learn to network. You learn to get in contact with people, you learn to reach out. So that helps.” (Natalie, 45, 10 Y.o.E) | **3b. Networking/reaching out for role models** |  |
| “The only thing would be the experience from other projects. Before you get too attached to what you could do yourself and then think about ideas that someone else might have already implemented, it’s better to ask another project or just have a look, no matter what the topic, whether it’s project management or content related because we didn’t really know how to do about it at the beginning. And before we have to exchange ideas with the project management agency several times and spend a lot of time and capacity internally to write something like that, we prefer to ask somewhere else where the focus is.” (Marie, 36, 2 Y.o.E) |  |  |
| “I am lucky in this project because the employees are very hard-working and very independent. But it is also a bit with the employees and how they work.” (Daisy, 46, 4 Y.o.E) | **3c. Having (the right) people to count on** |  |
| “If I had the choice, I would like to put another person in. So, to clone [colleague name], so to speak, she is top-notch. But she would be even better; simply in terms of the time we would then have. If another person, who works like her, could work for the project. We would have more personnel capacity. That would be wonderful.” (Queenie, 64, 22 Y.o.E) |  |  |
| “We complement each other perfectly. And that is just the beauty of it.” (Ulva, 55, 10 Y.o.E) |  |  |
| “In fact, the partners always come up with good suggestions for solutions. Some of them are very good at assessing themselves. After all, many partners are active in several projects and can perhaps look to the left and right.” (Karen, 30, 0 Y.o.E) |  |  |
| “The only challenge I can think of now is that you have to have the right people in your team, and I think I overreacted a bit, so it’s not so bad that that’s gone (laughs)...you don’t need employees who don’t know what they are supposed to do.” (Marie, 36, 2 Y.o.E) |  |  |
| “I also realize I make a lot of rookie mistakes. But as I said, when you have partners who see themselves a bit as companions, then you can also make a mistake.” (Moses, 56, 2 Y.o.E) |  |  |
| “... from project planning [laughs], where you simply learn a lot now. What you should have planned differently and what you should have applied for differently and in the project implementation.” (Claire, 40, 6 Y.o.E) | **4a. Building on experiences and (being open to) learning constantly/learning from mistakes** | **4. Self-growth and reflexivity** |
| “As a rule, this is the case: I’m in a project, and there are some changes, or the project brings a change. And then it changes a part of my life or my experience.” (Olivia, 29, 3 Y.o.E) |  |  |
| “Be open to the fact that you have to learn something.” (Walter, 58, 6 Y.o.E) |  |  |
| “I mean most people perceive that someone wanted to do something good, and he just did it a bit clumsily. So that is also a basis for clearing up such misunderstandings.” (Moses, 56, 2 Y.o.E) |  |  |
| “I also read communication psychology to get myself fit and also visualize processes by somehow applying the values square or something like that.” (Moses, 56, 2 Y.o.E) |  |  |
| “I do have experience from the municipal health conference. Even though I don’t manage money there, it goes in a similar direction, so it’s not that hardcore. I believe that when you have done several projects like this, you get a bit calmer and serenity into it. I get very excited about securing the whole budget and stuff like that. These are unfamiliar things to me, and then I get really excited. I have to say that quite honestly. It’s a challenge, and sometimes my heart beats faster (laughter)... I can well imagine, if we do a continuation or a new project, then I already know a bit more about it.” (Moses, 56, 2 Y.o.E) |  |  |
| “I’ve been working as a manager for several years now... in this context, you naturally take part in further training, further education.” (Tina, 55, 5 Y.o.E) | **4b. Taking part in further training and education** |  |
| “If you get support from human resources development, if that exists in the company, I will take it on at any time in the future.” (Marie, 36, 2 Y.o.E) |  |  |
| “It was a transnational project. You have to take cultural differences into account. You have to learn to deal with them because they might have different views or cultural backgrounds.” (Elaine, 42, 4 Y.o.E) | **4c. Learning-by-doing** |  |
| “If you get support from human resources development… I would take the [training] on at any time in the future. Unfortunately, it wasn’t like that for me. So, it was learning-by-doing.” (Marie, 36, 2 Y.o.E) |  |  |
| “I have a very open management style...My task was simply to guide, control and give the employees so much freedom that they could organize themselves and plan their activities.” (Anna, 53, 12 Y.o.E) | **4d. Being aware of own style, strengths, and weaknesses** |  |
| “When it comes to responding to individual needs, there is still something where there is probably still room for improvement.” (Olivia, 29, 3 Y.o.E) |  |  |
| “But you also have to look at which working method makes sense for you, especially in a fixed project with the project goal.” (Walter, 58, 6 Y.o.E) |  |  |
| “You are always the one who has to put a bit of pressure on [other project leaders] because you are sometimes the bad guy. Otherwise, how can I put it a bit more professionally? You are the one who has to keep up, and not everyone likes that. And that’s perfectly okay. But at that moment, you feel, you think, depending on what type of person you are. And I am very in need of harmony (laughs).” (Marie, 36, 2 Y.o.E) |  |  |
| “I try to develop a professional attitude (laughs), but of course, that doesn’t work; especially when they are not just occasional project partners but simply professional companions. And it’s sad when there’s tension and the nice cooperation suffers. Well, I am a person. Some people really work through their things purely factually and are hardly recognizable as a person. That is not my way. But I also believe that my way of bringing in a bit of my personality is not less efficient, maybe even a bit more efficient.” (Moses, 56, 2 Y.o.E) |  |  |
| “I also noticed that I had reached my limits in terms of communication. So, I had to learn foreign languages, so to speak, and had to ask a lot of questions: Explain this to me.” (Moses, 56, 2 Y.o.E) |  |  |
| “For me, the administration is not my strong point. And then you shouldn’t neglect it, but on the contrary, you should make sure that it works well through a good coalition with someone who can and may support it.” (Moses, 56, 2 Y.o.E) |  |  |
| “Look how you can promote skills so that our weaknesses… are not weak at the end or getting to strengths. I think it should be more like coordinating, I see me more like a coordinator than a leader.” (David, 42, 3 Y.o.E) |  |  |
| “Know your own strengths and your weaknesses and reflect on all the work, such as how you talk to each other. Reflecting and knowing your best style. Not only for managers, but I think it’s imperative for project leaders to know this.” (David, 42, 3 Y.o.E) |  |  |
| “It requires a high ability to reflect on one’s activity and oneself to lead such interdisciplinary projects.” (Anna, 53, 12 Y.o.E) | **4e. Being self-reflective/critical** |  |
| “Certainly, you have noticed that with certain things, what they actually do to you and what they have to do with you ... it’s often quite good to question yourself... whether those with whom wanted it to be completely different.” (Nelson, 46, 14 Y.o.E) |  |  |
| “It always gives you an opportunity to think about your self-image and your role models. And I believe that this is also important in these projects under the leadership aspect.” (Barry, 64, 9 Y.o.E) |  |  |
| “I think it’s absolutely crucial for a leader to maintain this kind of self-critical curiosity.” (Walter, 58, 6 Y.o.E) |  |  |
| “I need a tremendous amount of self-criticism for that.” (Walter, 58, 6 Y.o.E) |  |  |
| “Especially when you do something like this for the first time, you want to do it right, and you actually want to do it perfectly (laughs). And then, you also get frustrated and ask yourself, ‘what part do I have in it? Is it because I am not yet so well trained in project management? Maybe I still need something there? Is it because my network doesn’t work well? Or do I have a sufficient network? And where are the problems that you can’t prevent, that you simply have to live with it in the project?’ And that’s what kept me busy in the first year.” (Marie, 36, 2 Y.o.E) |  |  |
